# RAG genomic variation causes autoimmune diseases through specific structure-based mechanisms of enzyme dysregulation

**DOI:** 10.1016/j.isci.2023.108040

**Published:** 2023-09-27

**Authors:** Neshatul Haque, Tomoki Kawai, Brian D. Ratnasinghe, Jessica B. Wagenknecht, Raul Urrutia, Luigi D. Notarangelo, Michael T. Zimmermann

**Affiliations:** 1 Bioinformatics Research and Development Laboratory, Linda T. and John A. Mellowes Center for Genomic Sciences and Precision Medicine, Medical College of Wisconsin, Milwaukee, WI 53226, USA; 2 Laboratory of Clinical Immunology and Microbiology, National Institute of Allergy and Infectious Disease, National Institutes of Health, Bethesda, MD 20817, USA; 3 Department of Surgery, Medical College of Wisconsin, Milwaukee, WI 53226, USA; 4 Department of Biochemistry, Medical College of Wisconsin, Milwaukee, WI 53226, USA; 5 Clinical and Translational Sciences Institute, Medical College of Wisconsin, Milwaukee, WI 53226, USA

**Keywords:** Molecular modeling, Molecular structure, Genetics

## Abstract

Interpreting genetic changes observed in individual patients is a critical challenge. The array of immune deficiency syndromes is typically caused by genetic variation unique to individuals. Therefore, new approaches are needed to interpret functional variation and accelerate genomics interpretation. We constructed the first full-length structural model of human RAG recombinase across four functional states of the recombination process. We functionally tested 182 clinically observed RAG missense mutations. These experiments revealed dysfunction due to recombinase dysfunction and altered chromatin interactions. Structural modeling identified mechanical and energetic roles for each mutation. We built regression models for RAG1 (R^2^ = 0.91) and RAG2 (R^2^ = 0.97) to predict RAG activity changes. We applied our model to 711 additional RAG variants observed in population studies and identified a subset that may impair RAG function. Thus, we demonstrated a fundamental advance in the mechanistic interpretation of human genetic variations spanning from rare and undiagnosed diseases to population health.

## Highlights

• RAG has a modular structure facilitating mechanical functions of each domain
• Multi-state models explain the effects of more genetic variants than single models
• Conclusive structural mechanisms are proposed for 97.6% of IEI mutations
• Structural scores identify a subset of rare population variants that may affect RAG

## Graphical abstract

**Figure:**
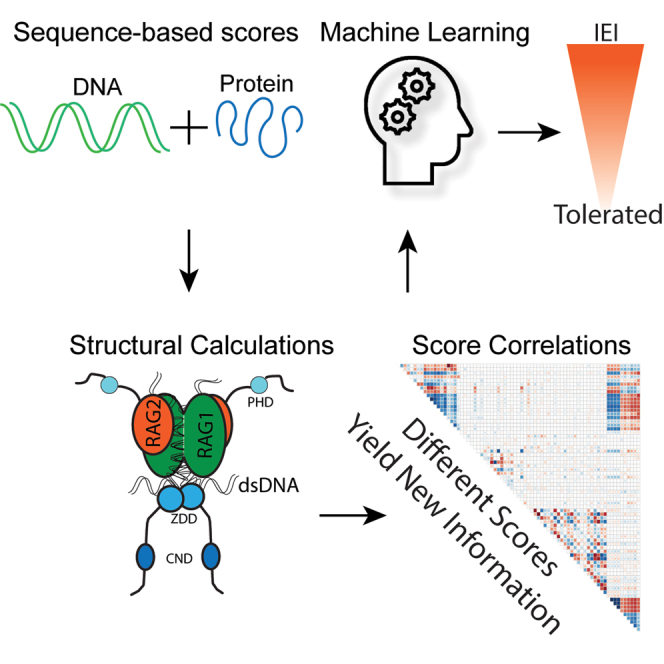

## Teaser

Molecular modelling; Molecular structure; Genetics

## Introduction

Clinical genomics sequencing is revolutionizing medicine through its ability to identify each patient’s genomic profile. New methods that can reveal mechanisms of disease and predict nuanced effects of individual genetic changes are highly needed. This need is acute across rare diseases and cancer where robust immune system functioning is key. Inborn errors of immunity (IEI) comprise more than 480 disorders, primarily due to pathogenic variants in genes involved in immune function.^1^ In addition to increased susceptibility to infections, immune dysregulation is also a common feature of IEI. In some cases, these two phenotypes result from variants in the same gene with opposed (loss-of-function versus gain-of-function) effects. However, a broad phenotypic spectrum may also be due to distinct hypomorphic variants (defined as causing incomplete loss of function), with different impacts on immune development and function on the one hand and immune tolerance on the other, as in the case of defects of the recombinase activating genes (RAG) 1 and RAG2.^1^^,^^2^ RAG1 and RAG2 are lymphoid-specific proteins that form a heterotetramer that initiates V(D)J recombination, ultimately enabling generation of a diversified T and B cell repertoire. Null *RAG1/2* variants cause severe combined immune deficiency with a lack of T and B cells (T- B- SCID). Hypomorphic RAG variants are associated with a variety of phenotypes that include Omenn syndrome (OS), atypical SCID (including expansion of γδ T cells), and delayed-onset combined immune deficiency with granuloma and autoimmunity (CID-G/A) and idiopathic CD4^+^ T cell lymphopenia.^1^ By using an *in vitro* recombination system, we have previously shown that the phenotypic spectrum of RAG deficiency is sustained by different degrees of functional activity of the *RAG* mutants, with SCID<OS<AS<CID-G/AI. However, the correlation is imperfect. Two further challenges remain: First, the underlying molecular mechanisms of dysfunction for known RAG variants are unknown, limiting research into possible interventions. Second, variants of unknown significance (VUS) in the *RAG* genes are often identified in patients with suspected immunodeficiency. Some of these are also present in population databases of presumably unaffected individuals. Further, evidence indicates that RAG alterations and aberrant function underlie the development of B and T cell cancers.^3^^,^^4^^,^^5^ New methods are needed to assess the impact of, and mechanisms for, genomic variation in RAG.

This study assesses genomic variation in the human RAG enzyme by combining data from across cell-based functional genomics assays, integrative 3D structural modeling, and protein structure-based calculations to mechanistically interpret a broad panel of RAG mutations. There is currently no experimental structure of the human RAG tetrameric complex. Thus, we leveraged integrative approaches to generate the first high-quality models, spanning four functional states required by the enzyme.^6^ We used the models to determine how genomic variants will likely impact RAG structure, in great mechanistic detail. Beyond the recombinase function of the core particle, other RAG domains are histone readers and function to target the enzyme to the correct regions of the genome. We identified mutations across different protein domains that impair RAG activity, and thereby dysregulate the immune system, due to altered structure of targeting and tethering functions, expanding IEI knowledge beyond direct effects on recombination. Then, we gathered experimental enzymatic activity values of 182 RAG1/2 variants. Further, we leverage our domain-centric observations and experimental data to produce a machine learning model for how protein structure-based changes due to IEI mutations will result in altered recombinase activity. Finally, we used the model to predict activity changes from population genetics databases. Machine learning enables statistical integration of diverse data, such as from our structural calculations with genomics data, to model their combined information. Interpretation of human variation in RAG to the level of mechanistic resolution we present herein was not previously possible. Thus, the current study is a significant advance in understanding the detailed changes to RAG that cause IEI and demonstrates the potential for broader adoption of similar methods for interpreting human genetic changes in Precision Medicine and Population Health initiatives.

## Results

### Molecular modeling of the human RAG complex using structural bioinformatics

Our interpretation of RAG genetic variants (Tables S1 and S2 contain enzymatic activity values and structural scores) extensively leveraged our understanding of the structural role of each amino acid within its respective protein domain, the flexibility of the domain required for recombinase mechanism, and the biochemical character of each region. A greater level of detail was necessary for us to understand the mechanistic role of each amino acid, their non-linear physicochemical interactions, and the effects of mutations. We succinctly summarize our structure-based interpretation of each RAG1 (Table S3) and RAG2 (Table S4) mutation supported by critical review of key structural features (Figures S3–S5). Overall, we find that RAG1 IEI mutations have a specific structural interpretation in 98.3% of cases, and RAG2 in 96.2% of cases. Thus, we provide the first of its kind, uniform, comprehensive structure-based mechanistic evaluation of IEI mutations in RAG.

### Structural components of the RAG1/2 complex and their characteristics

**Figure 1:**
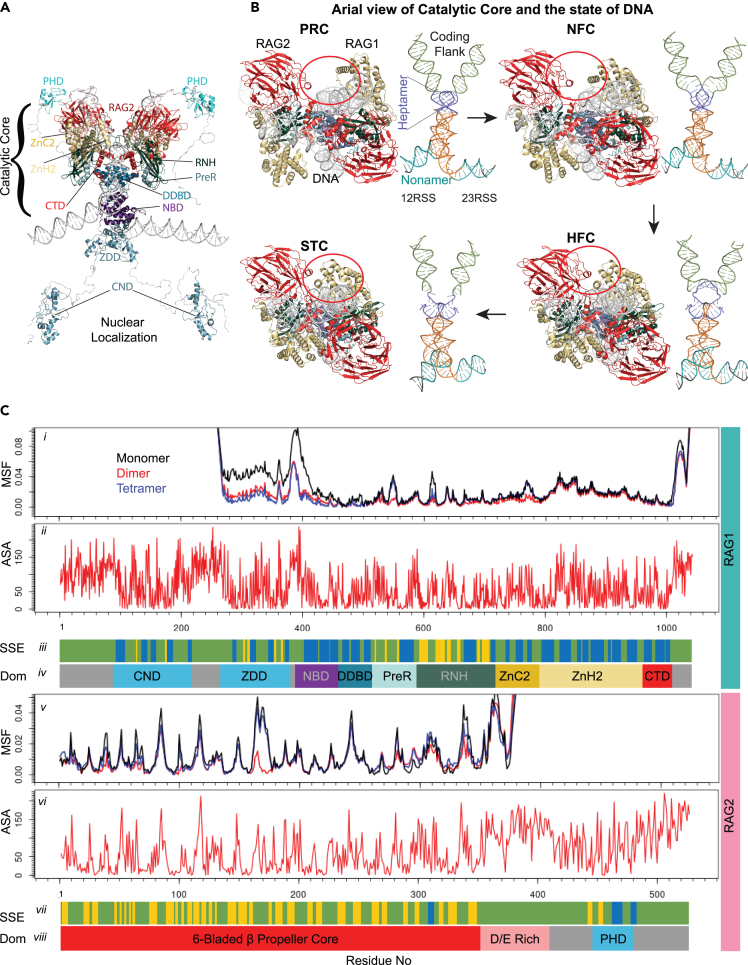
The first 3D model of full-length human RAG complex enhances the interpretation of genetic variation (A) We developed a full-length RAG complex model using an integrative approach. The catalytic core complex, RAG1 residue 387–1011, and RAG2 1–350 was developed using cryo-EM-generated PRC complex of mouse (PDB: 6OEM), RAG1 CND domain is modeled using AlphaFold2 predicted structure, RAG1 ZDD domain is modeled using mouse ZDD domain (1RMD), RAG2 PHD domain is modeled using mouse PHD domain (PDB: 2V88). The unstructured regions are included in the model for representation purposes only and have not been used in any analysis or drawing any inference. The DNA is nicked at the junction of coding flank and heptamer of RSS (see below), which is surrounded by RNH, ZnC2, and ZnH2. The first two helices of CTD interact with DNA and the third one interacts with DDBD. (B) We modeled the core in four functional states and highlighted different RAG1:RAG2 contacts that occur in each state, i.e., PRC (pre-reaction complex), NFC (nick-forming complex), HFC (hairpin-forming complex), and STC (strand transfer complex). The complexes are shown as an aerial view with respect to figure A and the associated state of DNA is shown as in figure A for proper visualization. Colors of domains are as mentioned in figure C *iv* and C *vii*; however, the components of DNA, such as coding flank, heptamer, and rest of the RSS, are shown in green, blue, and orange, respectively. (C) MSF of C-alpha atoms for RAG1 (*i*) and RAG2 (*v*), solvent-accessible surface area (ASA) (*ii* and *vi*), secondary structure elements (SSEs; *iii* and *vii*), and linear domain structure (*iv* and *viii*) for the PRC model.

Our model of the human RAG complex allows a new resolution of mechanistic investigation. RAG1 and RAG2 are multi-domain proteins (Figure 1A) that recognize well-conserved nonamer and heptamer recombination signal sequence (RSS) separated by less conserved 12 or 23 base pairs of DNA. For the enzyme to function perfectly, it is important to stabilize the substrate, the DNA nick site at the junction of heptamer, and coding region (Figure 1B).^7^^,^^8^^,^^9^^,^^10^ Our goal is to interpret IEI mutations using this new and highly specific mechanistic information. We detail this extensive information in a domain-centric manner through the following sections and in significantly greater detail in the supplemental results.

### Nonamer-binding domain couples structural and electrostatic supports

**Figure 2:**
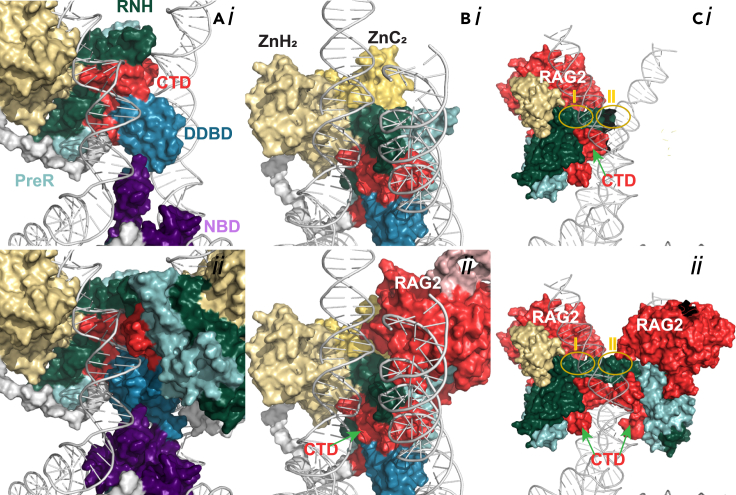
RAG1/2 domain and DNA surface interactions demonstrate necessary functional dynamics of PRC (A) The upper panel is RAG1 chain A surface interaction of NBD, DDBD, and CTD with the DNA from our model. The bottom panel shows the interaction of NBD, DDBD, and CTD of both the chains of RAG1 and one of the DNA. Colors are as in Figure 1. (B) The upper panel shows ZnC2 and ZnH2 interaction with DNA and the surrounding domains of RNH in the absence of RAG2 and bottom panel shows the same in the presence of RAG2. (C) PreR and RNH (work as a unit) do not extensively interact with DNA but at two places. In PRC complex, the active site residues (yellow ellipse 1) D603, D711, and E965 do not interact with DNA but they come very close in NFC. Few interactions were observed in the loop region L12 (yellow ellipse II) where, the backbone N of H612 makes hydrogen bonds with the other DNA strand. The lower panel shows PreR-RNH arrangement in the heterotetrameric complex.

**Figure 3:**
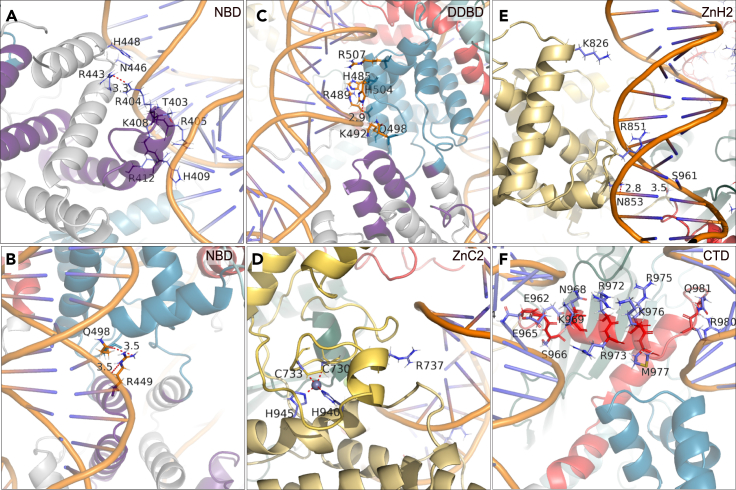
Residue-level interactions define important structural features that are altered by IEI mutations The interactions shown are from our PRC state model of the complex. (A and B) NBD: Along with the stable hydrophobic core, NBD shows electrostatic interactions with DNA and DDBD. (C) DDBD: The domain possesses a cluster of positive charged residue on the DNA interaction site of both the DNA helix. Holds the two DNA together. (D) ZnC2: The domain has only one arginine residue (R737) which is directed toward the DNA groove. This suggests that ZnC2 weakly interacts with DNA by electrostatic interaction. (E) ZnH2: The domain interacts with DNA from multiple locations. The residues N853 and S961 make strong hydrogen bond with negatively charged DNA backbone oxygen atom where the distances between the hydrogen bond donor and the acceptor are 2.8 and 3.5 Å, respectively. The residue R851 is partially embedded into the groove but does not make any H-bond and residue K826 also does not make any H-bond with any other residues as well as the DNA nucleotide but is directed toward the groove. The positively charged residues are electrostatically attracted to the groove. (F) CTD: It consists of three helices (D965-M977, C984-T998, and K1000-N1007). The coil at both the ends of first helices interacts with DNA. The residue Q981 forms a strong H-bond with nucleotide in the groove. Colors are as described in the Figure 1.

The nonamer-binding domain (NBD) of both RAG1 monomers forms a dimer in the pre-reaction complex (PRC) and interacts with the nonamer (nine conserved bases) region of RSS DNA. The dimerized NBD holds the two DNA fragments closely together.^9^^,^^10^ The N-terminal residues 389–400 are mostly solvent exposed, possess no secondary structure, and show large atomic fluctuations (Figures 1A, 1C, and 2A). The C-terminal region of NBD is a helical region with a stable hydrophobic core and polar/charged side chains solvated or DNA bound (Figures 1C*i*, and S1A) with distinct interactions in the major and minor groves, crossing between the two DNA strands (Figures 3A and 3B) (for detail see Results S1). Thus, the NBD comprises key structural and electrostatic features that support the tetrameric complex and DNA interactions.

### Dimerization and DNA-binding domain provides additional stability

The dimerization and DNA-binding domain (DDBD) is another hydrophobic core containing globular homodimer which holds the two DNA segments^11^ (Figures 1A, 2A, and S1B). DDBD monomers are comparatively less mobile than the dimer (Figure 1C*i*). Alpha helices are arranged in a crisscross manner, tapered at the bottom, and spread at the top. The DDBD N-terminal helix is connected to the NBD C terminus through a coiled region, and the C-terminal coiled region extends into the pre-RNase H (PreR) domain. The structural integrity of the DDBD plays a crucial role in holding the two DNA strands together with the help of its surface-exposed polar and positively charged residues. DDBD also makes electrostatic interactions with NBD near the major grove (Figure 3B). These positively charged residues anchor the less conserved region of DNA spanning between heptamer and nonamer regions (Figure 3C) (for detail see Results S2).

### The PreR domain forms a stable, structured coil configuration

**Figure 4:**
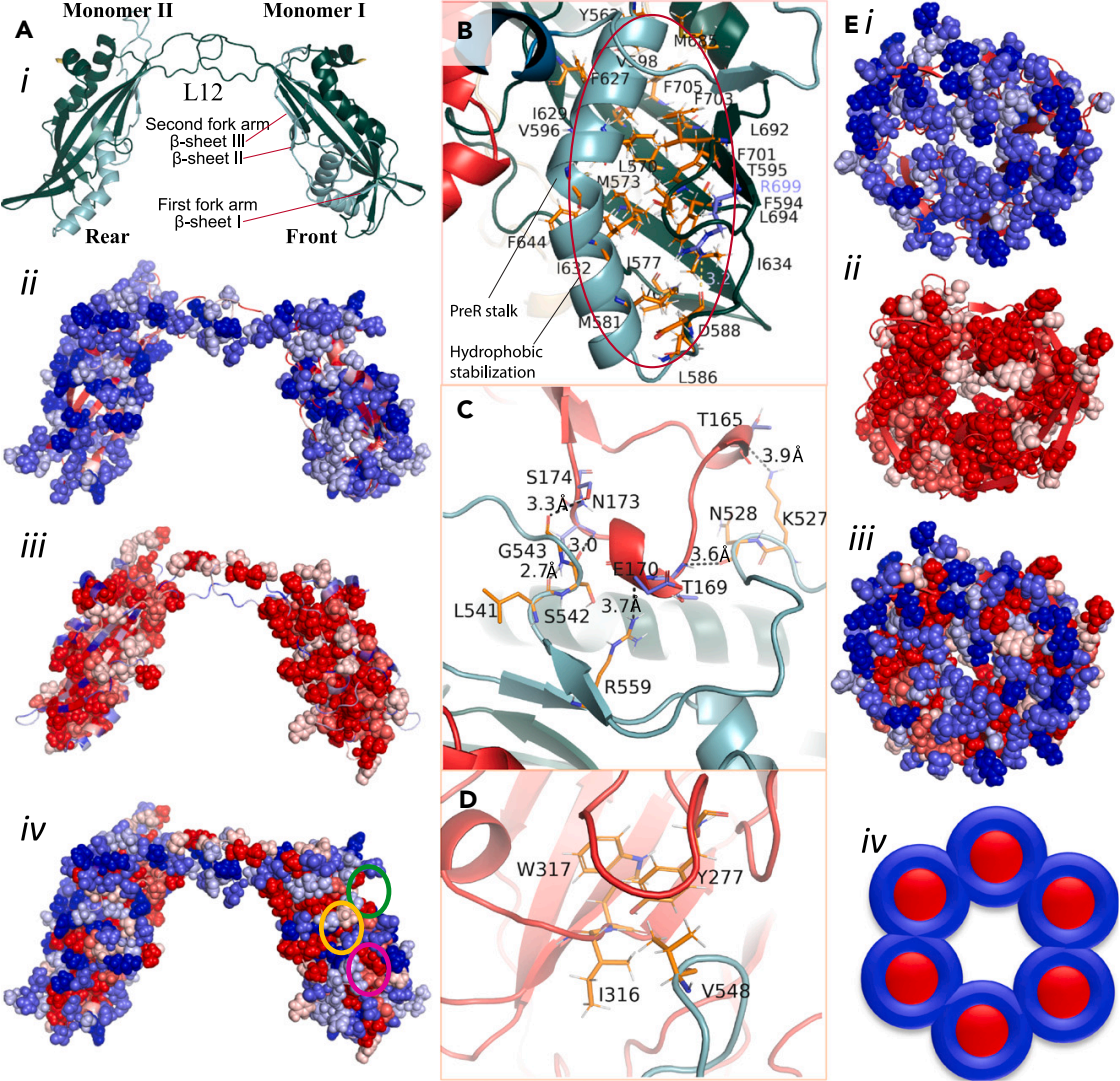
PreR stabilizes the RAG1/2 enzymatic core complex (Ai) The PreR-RNH heterodimers from our PRC state model are in a configuration resembling a hand shaking gesture via the L12 loop. The PreR (light cyan) and RNH (dark green) wrap around each other on the inner side of the core enzymatic complex. The β-sheet I (F520, E521, and W522) in first fork arm and β-sheet II (I537, I538, and D539) and β-sheet III (A557, K558, R559, and F560) in second fork arm extends into RNH main β-sheet structure. The hydrophilic (ii) residues are distributed on the surface and prevent the hydrophobic residues from solvent exposure (iii-iv). Few hydrophobic patches appear to be solvent exposed when observed in isolation but in complex they are important hydrophobic interaction point for other domains, such as, G707, G709, Y710, L714, V715, and V718 of RNH interact strongly with P786, F787, I788 of ZnC2 green circle, and yellow circle shows the interacting residues from 4B. (B) The stalk region of PreR in PreR-RNH complex is mostly stabilized by hydrophobic core, as shown by pink circle in A - iv. (C and D) There are many crucial electrostatic (C) and hydrophobic (D) interactions stabilizing PreP-RAG2 complex. (E) Spatial distribution of hydrophilic (i) and hydrophobic (ii) residues separately and together (iii) of RAG2 core. (iv) A simplified model of the hydrophobic and hydrophilic distribution.

Two units of the PreR domain are surrounded by RNase hybrid (RNH) (Figure 4A), RAG2 (Figure S2), and carboxy-terminal domain (CTD) domains (Figure 2C). PreR domain β-sheets are stabilized by extending into the β-sheet network of RNH (Figure 4A). The PreR stalk (Figure 4B) is made of a helix and two fork arm loops which are partly embedded in RAG2 and stabilized by specific charge-charge interactions and hydrophobic contacts (Figures 4C and 4D) The PreR domain and its RAG2-interacting loops have surprisingly low mobility for having <50% secondary structure content. The RAG2 loops that interact with PreR-RNH have low mobility (Figure 1C). However, the primary RAG2 core domain stabilization is caused by hydrophobic interaction (Figure 4E; details in Result S3).

### The RNH catalytic domain spatially coordinates the active site

RNH monomers come closer together near the junction of the coding flank and heptamer region of both the DNA strands in the PRC (Figure 1A). The PreR domain has a complementarity shape to the RNH making an extensive contacting surface area (Figures 2C and 4Ai), while also contacting the CTD, ZnC2, ZnH2, and RAG2 core domains (Figures 2B and 2C). Two active site residues come from RNH (D603 and D711), and the third from the CTD (E965)^12^^,^^13^^,^^14^ (Figure 2C). In the PRC, the active site residues are not aligned with the nick site of the coding sequence. The domain contains mostly β-sheet architecture and is stable across all four states of the recombinase cascade; the root-mean-square deviations (RMSDs) of the RNH (excluding L12) for nick-forming complex (NFC), hairpin-forming complex (HFC), and strand transfer complex (STC) states are 0.56, 0.66, and 0.66 Å, respectively (for detail see Results S4). In contrast, the L12 and L34 loops make new hydrogen bonds in HFC and STC, including the nicked DNA strands. Thus, the RNH domain has many active components that participate in enzymatic activity and stabilize the transition state.

### The PreR and RNH domains function as a cohesive structural unit

When observed individually, the PreR and RNH each have many surface-exposed hydrophobic residues (Figures S1C and S1D). However, when observed as one contiguous unit, very few hydrophobic residues are exposed (Figure 4A). So, it is reasonable to believe that both domains act as one structural unit having one continuous long cylindrical hydrophobic core wrapped around by hydrophilic residues. These hydrophilic residues interact with solvent, DNA, and other domains (for detail see Results S5).

### Adjacent zinc-binding domains coordinate with cysteine (ZnC2) and histidine (ZnH2)

ZnC2 domain has a classical zinc-finger architecture which coordinates Zn^2+^ with two Cys and two His residues (C730, C733, H940, and H945). It interacts with a conserved single-stranded DNA sequence, the heptamer RSS.^15^ The domain has <38% secondary structure (Figure 1C*iii*). Having less secondary structure and lack of hydrophobic core makes it more labile. Its major source of stabilization is its interaction with ZnH2, RNH, and RAG2 (Figure S1E; for detail see Results S6). In PRC, ZnC2 does not interact with RSS. However, its capability to interact with conserved sequence of non-B DNA^15^^,^^16^ suggests that its role could be crucial in the catalysis by stabilizing other states.

ZnH2 consists of eight short and long sequences of helices. The four long helices are nearly parallel to each other and make a cylindrical core. The four short helices are stationed outside the central cylindrical structure and comprise most of the RAG2^Trans^-interacting interface. Together, these two groups of helices make the domain a typical globular protein with a hydrophobic interior and polar and charged exterior. In the PRC, ZnH2 stabilizes the ZnC2 and substrate DNA near the heptamer region (Figure 2B). The core of the ZnH2 is also stabilized by Zn^2+^ coordination by C730, C733, H940, and H945 (Figure 3D). The loop between the α-helix II and III is embedded in the minor grove K826 facing the adjacent major groove (Figure 3E). The ZnH2 stabilizes transition states. The characteristic feature of the domain is its α-helical composition and a long continuous hydrophobic core, which makes it more flexible than other domains (Video S1). Because of the flexibility, the structure of the domain is slightly different from PRC in each state, with the RMSDs to NFC, HFC, and STC of 1.19, 1.32, and 1.15 Å, respectively. Its primary role is stabilizing the nicked DNA strands and the RAG2^Trans^ in HFC and STC (for detail see Results S7).

Video S1. Resilience in spring nature of helical ZnH2 domain, related to Figure 1 and Figure 2 This animation shows the high level of mechanical motion of the essential components of the complex, specifically the rearrangements within the ZnH2 domain across the recombinase cycle. While the domain moves as a semi-rigid body, there are many rearrangements of the amino acid side chains across the domain’s periphery. These rearrangements will present a different surface to the environment, to DNA, and to the other domains of the recombinase. Thus, loss of coordination of these movements due to mutations could dysregulate the enzyme. This animation supports our other figures for interpreting the role of each amino acid and the most likely effects of mutations. (See file, V1.MPG).

### CTD

The CTD consists of three helical regions, two with extensive DNA interaction and one with DDBD (Figure 1A). The CTDs’ coiled regions have substantial interaction with both DNA segments (Figures 3F and S6) (for detail see Results S8). The positively charged residues in the α-helix I are placed at maximum distance from each other in the PRC with the terminals of the helix locked between the two DNAs. The distances between the Cα-atom of the residue K976 of both the CTD are 12.0, 12.2, 14.4, and 14.6 Å in PRC through STC (Figure S6 and Video S2). A high level of mechanical motion is evident (Videos S3 and S4). Thus, a cluster of positively charged residues in such proximity with sequentially increasing distance suggests a rotatory motion, low friction motion analogous to diamagnetic repulsion in magnetically levitated trains, hinged on CTD while moving from one state to the other during the enzymatic process.

Video S2. Frictionless pivoting of RAG1/2 complex at CTD domain, related to Figure 1 and Figure 2 This animation shows the high level of mechanical motion of the essential components of the complex, specifically the sliding motion between monomers of the CTD. A distance monitor is shown as a dashed line connecting alpha carbon atoms to track the 2.6 Å shift. Multiple positively charged amino acids comprise this domain-domain interface. These charge-charge interactions should be repulsive; we interpret this organization like a magnetic lubrication where other interactions such as with DNA stabilize the overall enzyme while charge repulsion allows smooth motion within the CTDs. (See file, V2.MPG).

Video S3. High level view of RAG1/2 complex motion of domains and loop L12, related to Figure 1 and Figure 4 Animation showing the interpolation of the RAG core particle protein domains between the functional states modeled in this study. Domains are colored as in Figure 1. DNA is omitted for clarity. The twisting and sliding within domains are more visually evident compared to static images, such as in the stalk of the enzyme. Large scale motion of the loop L12is also evident. (See file, V3.MPG)TT.

Video S4. Arial view of RAG1/2 complex motion of domains and loop L12, related to Figure 1 and Figure 4 The same interpolation as in Video 1, we now show from a top-down perspective so the large-amplitude motion and changes in 3D contacts for RAG2 are easily visible. There are changes not only in RAG1-RAG2 contacts, but also flexible changes in inter-monomer RAG1 interactions. The video supports our visual and textual descriptions of these differences in the four states and crucial contacts made by loop L12. (See file, V4.MPG).

### Genetic mutations alter state-specific structural features

The role of all the residues for optimal catalysis of the huRAG1-RAG2-DNA complex cannot be understood by studying the inactive PRC complex. This is because the complex goes through many intermediate structural states, of which three have been characterized. These states require new interactions for stabilization so the enzymatic action can take place on the substrate. In this study, we have discovered residues that stabilize interactions necessary for optimal activity of the complex, across its enzymatic cycle.

### Nicked DNA stabilization

The role of the loop L34 could go beyond the stabilization of only the PRC complex by establishing the network of electrostatic interaction, as discussed in the previous section. It has been observed that loop L34 is involved in stabilizing the DNA strand at the nicked end (Figure S7A) in NFC and STC. The variant S651P may disrupt the electrostatic interaction network of loop L34, which eventually destabilizes the nicked DNA strand in HFC and NFC. The role of S651 is better understood by analyzing a 3D model of HFC and STC.

The activity of the variants T708A and G709S, 24.7% and 0.6%, could not be explained by studying the 3D model of PRC alone. This is because the residues T708 and G709, present at the C terminal of β-sheet VI, are near the loop L34 and interact with nicked DNA (Figure S7B). The H-bond between T708 and the backbone of the residue W959 (present between ZnH2 and CTD) is wholly lost in NFC, HFC, and STC. The mutation G709S may also disrupt the stability by introducing a new H-bond donor into an already balanced system. The loop containing the residue W959 is crucial for stabilizing the nicked DNA strand.

### Enhanced stability by loop L12

Loop L12 is one of the most dynamic regions (Videos S3 and S4) in the enzymatic core complex. The loop shows *trans* interaction with other DNA and ZnH2 in HFC and STC. The position and interactions of L12 are similar in PRC and NFC (Figure S8A) but significantly differs in HFC and STC (Figure S8B), and variants H612R and P619L possess higher activity (>120%) than wild-type RAG1. In the transition states, the residues such as H612, P619, and E610 show new interactions (Figures S8C, S8D, and S7F). The mutation H612R may increase stability because of stronger interaction made by arginine with DNA. The residue K847 and the mutation P619L might also add to the stability of the transition state by enhancing the hydrophobic interactions among V617, V618, and P619 with RNH residues L662 and A663 in HFC and STC state (Figure S8E and S8F). E610 stabilizes HFC and STC by making H-bond interaction with R841 (Figure S7F).

### Enzymatic activity prediction and classification leveraging integrated DNA sequence and 3D structural features

We calculated all-pairs correlation among sequence- and structure-based scores (Figures S9A and S9B). We found that experimental activity values overall correlated better with sequence-based scores than structure-based scores. Further, that correlations were higher for RAG2 variants than for RAG1, the maximum value of Pearson correlation is for the VEST score, both for RAG1 (0.553) and RAG2 (0.726) (Table S5). Other scores, including REVEL, VARITY, and esm1v, also perform well for RAG2. But for RAG1, only REVEL and PolyPhen2_HVAR could secure the Pearson correlation above 0.5. The cross-correlation matrix plot also suggests that the correlation between the activity and sequence-based scores significantly increases when the outliers are removed (Figures S10 and S11). Therefore, a better understanding of outliers could dramatically increase the correlation between the scores and activity, which could help develop a better prediction model.

The classification task of variants based on the experimental activity was performed using the pathogenicity probability of sequence-based scores (or sequence-based pathogenicity scores, SPS), as the activity and the prediction scores were well correlated. We analyzed the area under the receiver operating characteristic (ROC-AUC) curve for a two-class classification of activity profiles using the SPS. The activity values were marked as “0” and “1” for above and below a specific cutoff value. The cutoff value was scanned from 70% to 150% of activity, and ROC-AUC analysis was performed for all the sequence-based scores. It was observed that VEST4 SPS could classify the activity data into two classes with the maximum area under the curve for RAG1 (AUC = 0.917) as well as RAG2 (AUC = 0.946) (Figure S12). The ROC-AUC analysis reveals that the SPS of VEST4 for RAG1 can classify the variants as having activity below 89% of WT activity with 0.792 accuracy, and above that with 0.928 accuracy. Similarly, for RAG2, it was found that VEST4 can classify the variants to have activity above 91% of WT activity with 0.857 accuracy and below 96% activity with 0.960 accuracy. The classification result thus suggests that SPS possesses discriminatory power for variants.

**Figure 5:**
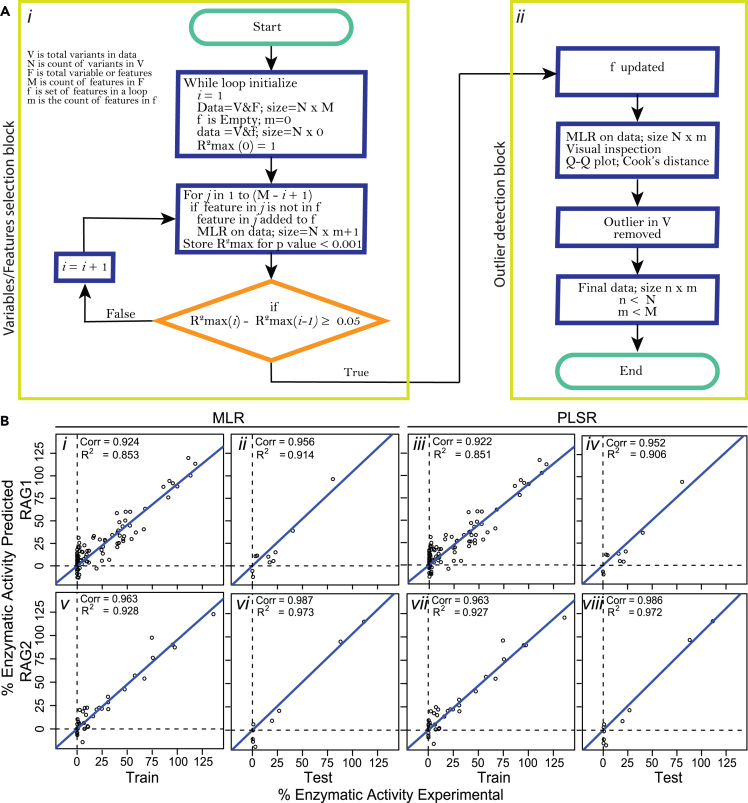
Regression models capture the observed variability in RAG activity changes due to mutations (A) Simplified workflow diagram of regression model development. (*i*) The variables or features selection and (*ii*) the outlier determination procedures are separately shown. (B) Performance of each model on training and testing datasets. (*i, iii*) The MLR and PLSR regression model for RAG1 train-test dataset (the seventh cross-validation fold is shown) demonstrate the feasibility for predicting mutation activities from 3D calculations. (*ii, iv*) Model performance was similar across cross-validation folds (the ninth is shown for comparison). (*v, vii*) RAG2 models were similarly strong with the MLR and PLSR regression model for training (fold nine shown), and (*vi, viii*) test set prediction. The regression model metric R^2^ for prediction of test data is depicted on each panel. Root-mean-square error (RMSE) for the prediction of test data for RAG1: MLR = 10.34%, PLSR = 10.01%; RAG2: MLR = 9.57%, PLSR = 9.03%.

We followed a specific procedure for constructing and training prediction models for RAG activity (Figure 5A). The datasets (containing scores and activity) of RAG1 and RAG2 were separately used for regression model development using multiple linear regression (MLR) and partial least-squares regression (PLSR). Variable selection was made using a forward approach wherein regression is first performed by using one predictor (i.e., scores), and the predictor which gets the best R^2^ value along with p value ≪ 0.001 is selected, and the next round of MLR is performed in the presence of previously selected predictor, and again the assessment was performed similarly. We finally obtained sets of variables with increasing values of R^2^. From this list, we selected that set of variables beyond which there is no significant increase in the R^2^ value. Following the step of variables selection, MLR is performed on the dataset, and the outlier variants (or observation) were determined using the suggestions of Cook’s distance and Q-Q plot. The entire process is repeated after excluding the outliers. For RAG1, the R^2^ value of 0.853 was obtained with 26 variables, 11 outliers, and 104 observations (Figures 5A*i*, 5B*i*, 5B*iii*; Tables S1 and S3), and for RAG2, the R^2^ value of 0.928 was obtained with 11 variables, six outliers, and 46 observations (Figures 5 A*ii*, 5B*v*, 5B*vii*; Tables S2 and S4). After variable selection and outlier removal, 20 different train-test datasets were created for RAG1 and RAG2 separately. In each dataset, ten random variants were withheld for testing the model and the rest were used to train the model using MLR and PLSR. The PLSR was performed with leave-one-out cross-validation to develop PLSR model on the selected variable and observation for the corresponding dataset. Finally, we developed 20 different models using MLR and 20 different models using PLSR for RAG1 and RAG2. Top models on each testing datasets were recorded for RAG1-MLR model as R^2^ = 0.914 (Figure 5B*ii*) and RMSE (root-mean-square error) = 10.34%, RAG1-PLSR (18 components) as R^2^ = 0.906 (Figure 5B*iv*) and RMSE = 10.01%, RAG2-MLR as R^2^ = 0.973 (Figure 5B*vi*) and RMSE = 9.57%, and RAG2-PLSR (6 components) as R^2^ = 0.972 (Figure 5B*viii*) and RMSE = 9.03%. Thus, we observe that for RAG1, more scores are required for considerable model building, suggesting a more complex and modularized nature of RAG1 than RAG2.

### Structural characteristics of outlier variants

In the regression studies, we have noted a few variants as outliers. The outlier variants of RAG1 are G325D, M435V, A456V, R474S, R474H, T477S, H612R, P619L, L836V, A957V, and M1006V. The outlier variants of RAG2 are V8I, T77N, P99Q, V145A, M322T, and G451A. We have observed that removing these variants from the model development improves the model’s accuracy and sensitivity. In general, we may conclude that these variants possess different characteristic chemistry than those represented by the 3D model. The 3D structural scores associated with these variants may be inappropriate, and they may attain different structural conformation than predicted by FoldX. A more rigorous dynamical study is required to obtain a better model for these variants, which is beyond the scope of this study.

### Prediction of functional effects for population genetic variation

**Figure 6:**
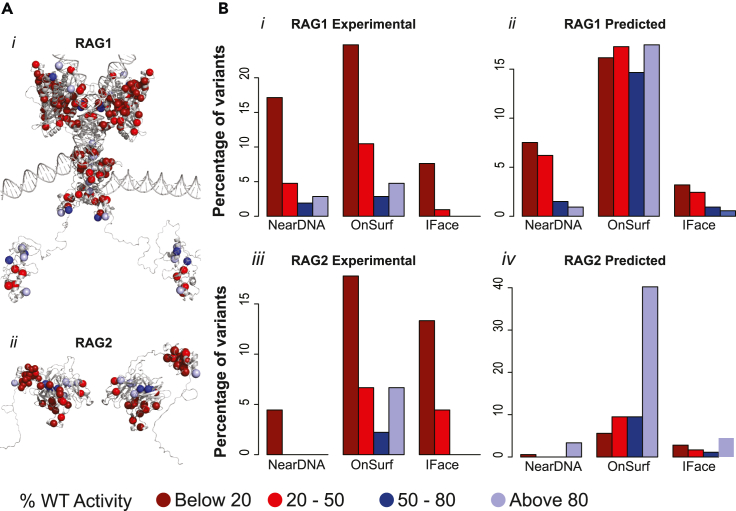
The distribution of variants across 3D model regions indicates IEI mutations and a fraction of population variations alter subunit interactions within the complex (A) 3D mapping of experimental variants (variants with experimental enzymatic assay data), shown as spheres, on huRAG-RAG2-DNA complex. Variants for RAG1 (i) and RAG2 (ii) are shown separately for clarity. (B) The distribution of variants in key location on the complex, such as near the DNA, on the surface of domain, and at the interface of RAG1-RAG2, are shown as they appear in the activity groups (AGs) like variants below 20% (AG1), between 20% and 50% (AG2), between 50% and 80% (AG3), and above 80% (AG4) of the WT activity of RAG1/2 complex. The variants for which experimental data are available in this study are marked as “Experimental” otherwise it is marked as “Predicted” in RAG1 and RAG2. The frequency of variants used in this plot is explained in Table S10.

We successfully applied our model, built on experimental data (Figure S13A and S13C; Table S6; and S8), to predict the recombinase activity of 711 variants, comprised of 532 variants in RAG1 (Figure S13B; and Table S7) and 179 variants in RAG2 (Figure S13D; and Table S9), genetic variations observed in population genetics studies (predicted scores are listed in Tables S6, S7, S8, and S9). These genetic variants obtained from different databases belong to various domains of the RAG1/2 complexes. The model for RAG1 and RAG2 has been used to predict the enzymatic activity of variants obtained from human mutation databases such as gnomAD, ClinVar, and more. There is a larger proportion of somatic mutation (e.g., cancer) in these databases compared to germline mutation for RAG1/2. Across distinct germline and somatic mutations, most were predicted to have lost their activity by our predictive model (Figure S13B). However, more than 50% of disease-associated variants were predicted to have activity above 80% in the RAG1/2 activity assay (Figure S13D). Variants proximal to DNA and at the RAG1/2 interface have a greater tendency to lose activity. However, we identified no clear activity association for population variants exposed to the solvent (Figure 6B), which contrasts what we observed for IEI mutations. This might be because of the smaller data size or large proportion of one type of variant which is cancer-associated variant. We thus identify a subset of rare population genetic variants that affect key features of the RAG complex, including DNA-binding interface and domain-domain interfaces, that define a subset of patients that do not have IEI, yet may have partially impaired RAG activity contributing to inter-individual immune response variation.

## Discussion

We present the broadest and most comprehensive to date, characterization of human RAG mutations associated with IEI, using a combination of laboratory experimental measurements and novel structure-based calculations. The data not only explain features of IEI mutations but also set up a process by which additional genetic variations from the general population can be scored for their likelihood of altering RAG function. We anticipate that this process can be repeated for additional human proteins and biomolecular complexes to significantly enhance the mechanistic interpretation of human genetic variation.

Most structural studies (experimental and bioinformatic) use one model of the protein encoded by each gene. In fact, the incredible recent advances in machine learning for predicting protein structures have produced proteome-scale databases of predicted structures, with one conformation for each un-liganded protein polymer. Yet, when possible, the most accurate modeling is knowledge-based curation with refinement. This is the approach we have used in the current work, wherein the function of RAG is not captured in any single conformation but must be modeled across multiple key steps in its enzymatic mechanism. Regions of RAG are likely to be intrinsically disordered and have a different role in the enzyme from structured domains. We have considered the local change in the energetic values to annotate mutations; unmodeled and intrinsically disordered regions do not affect our score calculations. Thus, our approach represents an expansion of structure-based study designs that projects genomic changes into the protein complex’s functional dynamics.

Our novel systematic approach to 3D structure-based scoring for interpreting RAG genomic variants identified from high-throughput sequencing^17^ applies to variants observed in cancer, germline diseases, and healthy-person germline sequencing. We have learned much about the WT mechanism and how it is altered in genetic diseases. This combined dataset provides insight into the RAG structure-function relationship, the fraction of VUS that may be functional, and mechanistic hypotheses for functional changes. Beyond the recombinase event, RAG domains are histone readers, and their activity is critical for applying the enzyme to the correct regions of the genome. Thus, the subset of mutations we identified as likely impairing chromatin interactions represents a different mode of immune dysregulation compared to inactivation of the recombinase. By identifying rare germline alleles resembling IEI mutations, we plan for future research to test which cause moderate RAG dysfunction that does not lead to IEI but may lead to slower adaptive responses or poorer functioning of the enzyme at different RSS sequences in the genome. Additionally, the aberrant activity of RAG, such as in B cell lymphoma, could be contributed by rare germline variation that damages the enzyme, predisposing individuals to later onset disease and inter-individual immune response variation. Therefore, the current study indicates this distinct possibility for further study, in addition to IEI mechanisms of dysfunction.

With the significant advance in resolution presented here, there remain IEI variants with similar activity to WT (≥90% WT activity): M324V, R449K, R474S, H612R, K820R, A868V, M1006V, and P1028L in RAG1 and V8I, P253R, F386L, N474S, and M502V in RAG2. These genetic variants may have effects when complexed with different RSS, modulate among the different IEI phenotypes, or alter interfaces with other substrates or molecular environments. Further, the effect of these missense alleles could be through changes to DNA or RNA structures, transcription factor binding, enhancer association, or yet other mechanisms. Thus, our addition of multi-state protein structure-based analyses is a critical layer of information to add to genomics but is not the only one we seek to encompass into a more advanced approach. Our future studies will expand our computational platform to additional biologic layers of information to build a more holistic understanding of RAG mutation function, population genomic effects, and how to translate this multi-scale and transdisciplinary modeling approach throughout the genome.

### Limitations of the study

We assessed functional activity to calibrate our computational model. Our *in vitro* assay to analyze the recombination activity of mutant RAG proteins has several limitations. Although wild-type human and mouse RAG proteins may combine to form functional heterotetramers, the functional impact of mutations perturbing protein complex association only in the autologous setting would not be readily identified by the *in vitro* assay. Furthermore, the inverted GFP cassette contained in the Abelson *Rag*^−/−^ pro-B cells is flanked by a single RSS pair. In contrast, at the immunoglobulin and T cell receptor loci, the RAG proteins sample many RSS that differ in their DNA sequence, allowing for stronger or weaker DNA cleavage activity across the set of sites. Therefore, the *in vitro* functional assay may not comprehensively assess the recombination activity of mutant RAG proteins. Overexpression of the mutant RAG protein may affect the interpretation of the results^18^; to mitigate this problem, *Rag*^−/−^ pro-B cell transduction with a RAG-expressing retroviral vector is conducted at a multiplicity of infection of less than 1 to minimize the occurrence of multiple integrations.^19^ Additionally, testing of the recombination activity has been typically carried out for one mutant at a time, but many patients are compound heterozygotes for two distinct *RAG* mutations. To circumvent this problem, bicistronic vectors expressing two mutant RAG proteins have been generated and tested in the experimental system.^20^ In conclusion, despite these limitations, the flow-cytometry-based assay represents a rapid and sufficiently robust way to analyze the recombination activity of naturally occurring human RAG mutant proteins. Our combination of experimental activity data and calculations across four states of structural models constitutes a clear step forward for interpreting the enzymatic effects of pathogenic and population-based genetic variation and their underlying damaging effects on the RAG protein complex.

### Conclusions

The RAG recombinase is sensitive to mutation. Such high sensitivity can be attributed to the modularized nature of the complex, where each module must perform its role precisely for stabilizing the DNA-RAG1/2 complex and its transition states before the enzyme breaks and rejoins the DNA. The present study demonstrates that structure-based calculations add information to sequence-based scores, enhancing the mechanistic interpretation of RAG mutations. Also, when appropriately combined with systematically derived weights, machine learning models predict recombinase activity values and classify variants as high or low activity. We found that PLSR outperformed multiple linear regression for the same set of features. Yet, across RAG, our structural bioinformatics approach provided a mechanistic interpretation for 97.6% of IEI variants within structured domains. This level of resolution is nearly unprecedented for statistical methods. Therefore, we have significantly increased the resolution available for interpreting IEI mutations across RAG. For the genomics field, we have demonstrated the potential avenues for scaling structure-based methods to thousands of mutations. In summary, understanding functions requires context. We can functionally interpret human genomic variation by accounting for sufficient resolution of the multi-protein enzyme in the right environment and across dynamic functional states.

## STAR★Methods

### Key resources table

**Table:** 

REAGENT or RESOURCE	SOURCE	IDENTIFIER
**Data**		

Experimental Recombinase Activity Levels	Result of this study	Tables S1 and S2
Calculated RAG Complex Mutational Scores	Result of this study	Tables S1 and S2
Predicted RAG Activity	Result of this study	Tables S6, S7, S8, and S9

**Software and algorithm**		

BioR	Kocher, Jean-Pierre A. et al. 2014	https://bioinformaticstools.mayo.edu/research/bior/
dbNFSP	Liu X. et al. 2015	https://sites.google.com/site/jpopgen/dbNSFP
VARITY	Wu Y. et al. 2021	http://varity.varianteffect.org/
Ems-1v	Joshua Meier et al. 2021	https://github.com/facebookresearch/esm
FoldX	Joost Schymkowitz et al. 2015	https://foldxsuite.crg.eu/
FrustratometeR	Atilio O Rausch et al. 2021	https://github.com/proteinphysiologylab/frustratometeR

### Resource availability

#### Lead contact

Any information or request regarding the resources, raw data, and code will be fulfilled by Michael T. Zimmermann (mtzimmermann@mcw.edu).

#### Materials availability

Not applicable.

### Experimental model and study participant details

Not applicable to this study.

### Method details

In this study, we leveraged homology-based methodologies and integrative analyses for developing 3D models of the human RAG1-RAG2-DNA complex. We used these models to calculate relationships among mutated residues, predominantly observed in human immune diseases, and their role in enzymatic activity as measured by reporter assay. We generated predictive models of enzyme function leveraging the combined data across experimental measurements and 3D calculations.

#### Gathering observed human variations in the RAG complex

We generated two datasets of human genetic variation in the RAG complex. The first is a clinical dataset observed in immunodeficient patients, and the second is a broader cohort of human genetic alterations. To define the first set of genetic variants, anonymized clinical, immunologic, and molecular data of *RAG*-mutated patients were provided by an international network of physicians in Europe, the Middle East, South America, Asia, and the United States, according to protocols NCT03394053 and NCT03610802 approved by the NIH IRB. Variants were evaluated using our *in vitro* recombination activity assay.^19^^,^^20^ To define the second set of genetic variants, we gathered all observed human variants of *RAG1* and *RAG2* and their corresponding clinical significance annotations (when available) from four databases: gnomAD^21^ for public population alleles among generally healthy adults, HGMD^22^ and ClinVar^23^ for heritable diseases, and COSMIC^24^^,^^25^ for cancer mutations, using the BioR annotation platform.^26^ The genetic variants in our first set also appear in the second database set. We made a final list of variants after filtering out the variants present in intrinsically disordered or unstructured regions. The final counts for RAG1 are 647 variants, of which we measured experimental enzymatic values for 115 (Tables S1 and S3) The final counts for RAG2 is 231 variants, of which we measured experimental enzymatic values for 52 (Tables S2 and S4). Thus, we aim to characterize most alterations observed across a spectrum of human disease and non-disease contexts.

#### Sequence-based genomics pathogenicity scores

We used dbNSFP^27^ to gather sequence-based pathogenicity scores for genomic variants in our two datasets, which comprised 822 missense genetic variants (including variants in the unstructured region) of RAG1 and 455 (including variants in the unstructured region) of RAG2, with 22 distinct sequence-based prediction scores. Two additional types of sequence-based scores were calculated: VARITY, a machine learning (ML) model developed on Gradient tree boosting,^28^ and protein language model based esmv1 (evolutionary scale modeling v1).^29^ There are four types of VARITY models - two for rare variants (minor allele frequency, MAF <0.5%) and two for extremely rare variants (0 allele count in gnomAD) prediction. Each of the two is further divided based on two different validation methods. There are five types of esmv1 models, which are all MSA transformer-based protein language models. The score was calculated locally on PyTorch-enabled GPU-accelerated machines. We implemented a zero-shot learning approach for esmv1 variant score calculations (Figure S14).

#### Developing a 3D model of the human RAG complex in four functional states

V(D)J recombination is an intricate process that includes four significant enzymatic steps. The four major steps have been named, and their corresponding structures are determined for mouse RAG1-RAG2 recombinase.^30^ The recombination process initiates with the assembly of two monomers of RAG1 (1043 amino acids), two monomer of RAG2 (527 amino acids), and the two-recombination signal sequence (RSS) regions of DNA, designated the 12/23 RSSs containing 12 bp and 23 bp spacer sequences, respectively. This enzymatically inactive stage of the complex is named the Pre-Reaction complex (PRC). The PRC stalk is strengthened by the nonamer binding domain (NBD) and dimerization and DNA binding domain (DDBD) of RAG1. Before one of the DNA strands is nicked, it is unwound by rotating the coding flank, the portion of DNA containing the coding segment (Figure 1A), by180°, making it appear like a ladder.^30^ At this stage, the complex is ready to introduce the nick, called the nick-forming complex (NFC) (Figure 1B). After both strands of DNA are nicked, some rearrangements in the RAG1-RAG2 are observed, and a hairpin-forming complex (HFC) (Figure 1B) is attained. The HFC state is followed by strand transfer complex (STC) (Figure 1B). This transition also requires rearrangements in RAG1-RAG2 side chains and loops. Thus, data exists to generate models of the human RAG core complex in all four major states.

Structures of the enzymatic core complex from mice, rats, and zebrafish exist and were leveraged to construct models of the analogous human enzyme.^30^^,^^31^ We used homology modeling (modeler v10.1^32^) to develop the 3D structure of the human RAG1-RAG2-DNA complex using the murine experimental PRC core complex structure (PDB: 6oem; RAG1 sequence identity 90% (936/1043), positive 94% (984/1043) and gap 0%(3/1043) and RAG2 sequence identity 88% (466/527), positive 94% (500/527) and gap 0%(3/527); Figure 1A). The core domain (RAG1 residue 387–1011 and RAG2 residue 1–350) states were developed (Figure 1B) using murine templates: NFC, PDB: 6oeo (CryoEM, 3.6 Å)^33^; HFC, PDB: 5ze0 (X-ray, 2.75 Å)^34^; and STC, PDB: 6oet (CryoEM, 3.4 Å).^33^

The function of the non-core domains remains partly elusive. However, studies suggest that the CND acts in nuclear localization, zinc binding, and double-stranded DNA binding^35^ and PHD domains for tethering the enzyme to its primary chromatin substrate. The central non-core domain (CND, aa 87–217) of huRAG1 was modeled from aa 92–220 using AlphaFold2^36^ predicted structure. The pLDDT for huRAG1 aa 92–220 was of acceptable confidence. The model suggests that the residues C111, C114, C176, and C179 are involved in Zn coordination in CND. The huRAG1 Zinc dimerization domain (ZDD, aa 268–380) was modeled using the 2.1 Å mRAG1 ZDD dimer experimental structure, PDB: 1RMD. We docked our ZDD dimer model to the enzymatic core complex, using HDOCK server.^37^ The huRAG2 non-canonical plant homeodomain finger (PHD, aa 446–481) was modeled using the experimental structure of extended mRAG2 PHD domain finger (aa 410–480, PDB: 2V88 with 2.0 Å resolution). The X-ray structure mRAG2 PHD domain was co-crystalized with methylated arginine and lysine and was kept in the huRAG2 PHD modeling. The 12/23 RSS DNA 3D structure of the enzymatic core complex of the mRAG1-RAG2-DNA complex was preserved during the huRAG1-RAG2-DNA modeling. The stretches of sequences in RAG1 and RAG2 for which the template did not exist were left unmodeled in a coiled-coil manner (Figure 1A). In this way, we generated the first model of the full-length RAG heterotetramer complex to better interpret the effects of human genetic variants in this critical immunologic process.

#### Snapshot interpolation between the states

Transition snapshot of the 3D model from one state to the other for some domain such as shown in supplemental videos V1, V2, V3, and V4 are interpolated trajectories between the states and were computed using morph function of pymol.^38^ PyMol is also used for all the protein related graphics. These interpolated trajectories are not accurate, and we have not used these dynamics for any calculation but only to visualize the transition between the states as continuous.

#### Structural calculations for defining the effect of genomics on 3D protein structure

Structural calculation such as, secondary structure content, solvent accessible surface area were calculated using STRIDE^39^ and mean square fluctuation was calculated for RAG1/RAG2 monomer, RAG1/RAG2 dimer and the RAG1/RAG2 heterotetramer using Gaussian Network Models (GNM) as implemented in the python package ProDy. GNM is computationally less intensive with respect to molecular dynamics simulation and provides low frequency motion associated with the residues.^40^All RAG1 and RAG2 variants were modeled using FoldX v5.0.^41^ Sixteen different energy scores were also calculated using the 3D mutant protein models generated by FoldX. The loss or gain of local residue stability upon mutation in the protein was assessed using Frustratometer via the Frustratometer2 R package.^42^ Frustratometer2 calculates stability using three different methods: configurational, mutational, and single residue. We computed 21 derived scores from Frustratometer results, such as, differences in frustration and energetic stability, of the residue under study like variants residue, with respect to the wild type. The energy values and frustration indices were summed for the mutated residue, along with their differences from the wild type. Following these strategies, we generated a dataset of 68 sequence and structure-based scores (Tables S1 and S2).

#### *In vitro* functional characterization of RAG mutation activity

We used our *in vitro* recombinase activity assay to determine the enzymatic activity level of each RAG1/2 variant.^19^^,^^20^ In brief, the cDNA of each *RAG1* or *RAG2* variant was transduced into a RAG1^−/−^ or RAG2^−/−^ murine pro-B-cell line containing an inverted GFP sequence flanked by RSS. The sequence of 12 bp spacer RSS is TCC AGT CTG TAG. The sequence of 23 bp spacer RSS is GTA GTA CTC CAC TGT CTG GCT GT. Upon stimulation with STI-571/imatinib (Novartis, Switzerland) for 96 h to maintain cells in G0/G1, the recombination activity of the enzyme was measured in triplicate by flow cytometry by analyzing the proportion of GFP-expressing cells. Results were normalized to the proportion of GFP-expressing cells reconstituted with wild-type RAG1 or RAG2. The percent activity was reported as the replicate average.

#### Exploring the two-class classification capability of sequence-based scores

Sequence-based scores derived from dbNFSP are more correlated to the experimental activity values (Figures S9–S11). However, the best correlation achieved is 0.52 Spearman and 0.72 Pearson correlation for VEST4_S in RAG2 and 0.43 Spearman and 0.55 Pearson correlation for VEST4_S in RAG1 (Table S5). To search for the best classifier, we computed Area under the Receiver operating characteristic (ROC) curve (AUC) for sequence-based scores and computed the confusion matrix. To set the activity cut-off we first scanned the AUC with shifting activity value from 50% to 100% (maximum enzymatic activity value is 124 for RAG1 and 136 for RAG2). Following cut-off scanning for maximum AUC we computed the confusion matrix for that cut-off enzymatic value (Figure S12).

#### Variable selection and regression model development

The experimental enzymatic data for 125 (10 of the variants are present in unstructured region) mutations in RAG1 and 57 (5 of the variants are present in unstructured region) mutations in RAG2 were used for regression and two-class categorical prediction model development. The mutational scan for the activity of the huRAG1-RAG2-DNA complex was performed to understand the role of readily observed mutation and the function of the complex. We performed multiple linear regression (MLR) and partial least squares regression (PLSR) using experimental activity data as the outcome and structure-based scores as dependent variables. We found the optimal scores for generating the MLR model by iteratively adding scores to the model.

The datasets (containing scores & activity) of RAG1 and RAG2 were separately used for regression model development using multiple linear regression (MLR) and partial least squares regression (PLSR). Variable selection was made using a forward approach wherein regression is first performed by using one predictor (i.e., scores), and the predictor which gets the best R2 value along with p value ≪ 0.001 is selected, and the next round of MLR is performed in the presence of previously selected predictor, and again the assessment was performed similarly. We finally obtained sets of variables with increasing values of R^2^. From this list, we selected that set of variables beyond which there is no significant increase in the R^2^ value. Following the step of variables selection, MLR is performed on the dataset, and the outlier variants (or observation) were determined using the suggestions of Cook’s distance and Q-Q plot. The entire process is repeated after excluding the outliers. For RAG1, the R^2^ value of 0.853 was obtained with 26 variables, 11 outliers, and 94 observations (Figure 5B*i*), and for RAG2, the R^2^ value of 0.928 was obtained with 11 variables, six outliers, and 46 observations (Figure 5B*v*). After variable selection and outlier removal, 20 different train-test datasets were created for RAG1 and RAG2 separately. In each dataset, ten random variants were withheld for testing the model, and the rest were used to train the model using MLR and PLSR. The PLSR was performed with LOO cross-validation to develop a more robust model than MLR for the corresponding dataset. Finally, we developed 20 different models using MLR and 20 different models using PLSR for RAG1 and RAG2. A detailed step by step procedure of regression model development is shown as a workflow diagram (Figure 5A).

### Quantification and statistical analysis

For feature selection Cook’s distance and Q-Q plot analysis was performed (discussed in the result section, Enzymatic activity prediction and classification leveraging integrated DNA sequence and 3D structural features and Figure 5A). For testing the accuracy of multiple linear regression model and partial least squares regression model we employed root-mean-square error analysis, R^2^, and Pearson correlation (Figure 5B).

## Data Availability

• Data generated during this study is mentioned in the key resources table. • Code is deposited at https://github.com/neshatul/RAGactivityPred and is publicly available. • Statistical models are deposited at https://github.com/neshatul/RAGactivityPred and is publicly available.

## Supplemental information

Document S1. Figures S1−S14, Tables S5, S10, and Results S1−S8

Table S1. All computational and experimental scores used for characterizing RAG1 IEI genetic variations, related to Star Method and Figure 5 (See file, Table_S1_RAG1_Scores_125_74.xlsx)

Table S2. All computational and experimental scores used for characterizing RAG2 IEI genetic variations, related to Star Method and Figure 5 (See file, Table_S2_RAG2_Scores_57_74.xlsx)

Table S3. Structure-based mechanistic characterization of all studied RAG1 IEI genetic variations, related to Figure 6 (See file, Table_S3-RAG1_variants_explained.xlsx)

Table S4. Structure-based mechanistic characterization of all studied RAG2 IEI genetic variations, related to Figure 6 (See file, Table_S3-RAG2_variants_explained.xlsx)

Table S6. Experimental activity measures for all studied RAG1 genetic variations from IEI and population-based resources, related to Figure 5 and Figure 6 (See file, Table_S6-RAG1_Experimental.xlsx)

Table S7. Predicted activity measures for all studied RAG1 genetic variations from IEI and population-based resources, related to Figure 5 and Figure 6 (See file, Table_S7-RAG1_Predicted.xlsx)

Table S8. Experimental activity measures for all studied RAG2 genetic variations from IEI and population-based resources, related to Figure 5 and Figure 6 (See file, Table_S8-RAG2_Experimental.xlsx)

Table S9. Predicted activity measures for all studied RAG2 genetic variations from IEI and population-based resources, related to Figure 5 and Figure 6 (See file, Table_S9-RAG1_Predicted.xlsx)
