# Supplementary material for: RAG genomic variation causes autoimmune diseases through specific structure-based mechanisms of enzyme dysregulation
Source: iScience. 2023 Sep 27;26(10):108040. doi: 10.1016/j.isci.2023.108040 (PMC10579426; doi:10.1016/j.isci.2023.108040)
Supplement: Document S1. Figures S1−S14, Tables S5, S10, and Results S1−S8 [file mmc1.pdf]

iScience, Volume *26*

Supplemental information

RAG genomic variation causes autoimmune diseases through specific structure-based mechanisms of enzyme dysregulation

Neshatul Haque, Tomoki Kawai, Brian D. Ratnasinghe, Jessica B. Wagenknecht, Raul Urrutia, Luigi D. Notarangelo, and Michael T. Zimmermann

Supplemental Results

Result S1: NBD anchors the DNA substrate to the RAG1/2 complex, related to Figure 1.

The NBD of both the chains of RAG1 are dimerized in the pre-reaction complex (PRC) and interact with the nonamer (nine conserved base pairs) region of RSS DNA. The dimerized NBD, residue 389-459 from both monomers of RAG1 hold the two DNA fragments, closely together in the stalk of the Y-shaped complex. The N-terminal residues 389-400, are mostly solvent-exposed, possess no secondary structure, and show large atomic fluctuations (**Figure 1A, 1C and 2A**). Among them are three positively charged residues: K391, R394 and R396. Three hydrophobic residues are present in this region I389, L399 and L400 along with G392, G393 and P395, where L399 has a completely buried side chain whereas I389 and L400 are exposed to the solvent (**Figure S4 I**). The C-terminal region of NBD is a stable region where three helices (α-helix I R404-E426, α-helix II D429-R445 and α-helix III E447-Q459) from each RAG1 chain are stacked over one another to make a stable hydrophobic core and polar/charged sidechains are exposed to solvent or DNA binding (**Figure 1A**). The side chains of N446 and H448, at the turn between two helices, are partially embedded in one of the DNA minor grooves making polar interactions. A cluster of positively charged amino acids, R404, R405, K408, H409 and R412 of one monomer, is present at the adjacent major groove of DNA that also interact electrostatically with nucleotides and the phosphate backbone of the groove, except R404, which makes strong hydrogen bond with R443 of the other monomer (**Figure 3A**). Thus, the NBD comprises key structural and electrostatic features that support the tetrameric complex and DNA interactions.

Result S2: DDBD stabilizes the DNA to the RAG1/2 complex by interacting with less conserved region, related to Figure 1.

The DDBD is another hydrophobic core containing globular protein like domain, which is a homodimer and holds the two DNA segments in close proximity at the neck of the Y-shaped complex (**Figure 1A and 2A**). Two sides link to the CTD. The monomer of DDBD show comparatively less MSF than the dimer (**Figure 1C**). The DDBD domain contains mostly helices and is very stable because of the hydrophobic core. The helices are arranged in a cris-cross manner, tapered at the bottom and spread at the top. The DDBD N-terminal helix (α-Helix I P467-T477) is connected to C-terminal of NBD domain through a coiled region (G460-Q466). Other secondary structure elements followed by the first helix are: coiled region of three residues, F478, L479 and S480, helix (α-Helix II C481-T495); coiled region of seven residues, G496-P502; helix (α-Helix III L503-L513); and, the coiled C-terminal (L514-P515) region, which extends into PreR domain. The structural integrity of the DDBD plays a crucial role in holding the two DNA strands together with the help of its surface exposed polar and positively charged residues. The positively charged residues H485, R489, K492, H504, and R507 are partially embedded in the DNA major groove and the polar residue Q498 is placed near the backbone of DNA which is engaged in

hydrogen bonding (H-bond) interaction with R449 of NBD. These positively charged residue anchors both the DNA with DDBD (**Figure 3C**).

Result S3: Structural details of PreR domain, related to Figure 2 and Figure 4. Two units of the PreR domain are surrounded by RNH, RAG2, and CTD domains (**Figure 2C**). PreR is comprised of 14% beta-sheet, 31% alpha-helix, and 55% turs or coils, and is shaped like a tuning fork. First fork arm is from residue 520-534, second fork arm is from residue 536-561, and the PreR stalk is from 562-585 aa. (**Figure 4A and S2**). Its stable stalk region is made of helix and the two fork arms are mostly made of coiled coil, which are partly embedded in RAG2. The entire PreR domain is physically supported by RNH domain, except for part of fork arms embedded in the RAG2 (**Figure 4B and 4D**). The residues F520, E521, and W522 (β-sheet I) in the first fork arm and I537, I538 and D539 (β-sheet II), A557, K558, R559, and F560 (β-sheet III) in the second fork arm, participate in β-sheet of RNH, further strengthening the tuning fork. The domain emerges from DDBD and sets the secondary structure of its initial residues L514, P515, G516, and H518 (the first two residues are considered part of DDBD) as turns (**Figure S3 A**) to provide the residues F520-W522 an appropriate configuration which can subsequently make a stable β-sheet of PreR, This turn is crucial for the stability of PreR β-sheet as the variant G516A, which might destabilize the turn, loses 60% of the activity (Table S1). The PreR domain has surprisingly low MSF despite less than 50% of secondary structure content. Major dynamics in the PreR domain is observed around the residue S530 (first fork arm), around the residue V548 (second fork arm, most dynamic) (**Figure 1C**). The PreR stalk, which is mostly helix residue 562-585, is hydrophobically stabilized by RNH and the coil region in the C-terminal of PreR, residue D585-D588, which extends further to N-terminal of RNH, residue D588-P593 (**Figure 4B**). The tip of these fork arms is stabilized by interaction with RAG2 (**Figure 4C and 4D**). The first fork arm is stabilized by the intra-domain H-bond between residues R561 and S530 of PreR and inter-domain H-bond between the backbone of residues N528 of PreR & T168 of RAG2 (**Figure 4C**).

Result S4: Majority of RNH domain remains stable in all four states, related to Figure 2.

Two monomer's RNH domains come near the junction of the coding flank and heptamer region of both the DNA strands in the PRC complex (**Figure 1A**). The PreR domain straddles around RNH in a shape complementarity fashion which accounts for the largest surface area of contact of the domain. Other domains, which interact with RNH, are the CTD domain, ZnC2, ZnH2, and RAG2 core domain (**Figure 2B and 2C**). Two of the active site residues come from RNH (D603 and D711), and the third residue comes from the CTD (E965). In the PRC complex, the active site residues are not in close contact with the nick site of the coding sequence (**Figure 2C**). The domain has a high fraction of secondary structures with 43% of β-sheet, 21% of the helix, and 36% of turns and coils. The main framework is made of β-sheet that spans the length of the domain starting from β-sheet I (F594-D607), followed by β-sheet II (K621-A635), β-sheet III (N640-E645), β-sheet IV (K656-M661), helix I (H668-K686), β-sheet V (L690-E693), β-sheet VI (L698-G709) and finally ends in helix II (E712-E719) (**Figure**

1C iii). The most dynamic region in the RNH domain are the loops between the β-sheet I and β-sheet II, called L12. The loop L12 is primarily a non-hydrophobic residue containing loop that interacts with loop $L12^{Trans}$ and DNA^{Trans}, the loop and DNA of other RAG12 heterodimer. Such a polar and charged long loop has great potential to interact with distant charged residues and DNA nucleotides. The loop L34 (the loop between β-sheet III and β-sheet IV), consisting of 10 residues, is among the least dynamic region in RNH that interacts with CTD by H-bond, E652-S966 (**Figure S3 G**), but its role remains obscure with ZnH2 despite being close to each other in the PRC complex. However, it has been observed that this loop possesses an identical configuration in NFC, HFC, and STC with respect to PRC. The RMSD of RNH (minus L12) domain for NFC, HFC, and STC states are 0.562, 0.657 and 0.657 respectively. The loop L34 makes new H-bond contacts in HFC and STC with other residues as well as the nicked DNA strands. The RNH domain has many active components that participate in enzymatic activity as well as provide stability to the transition state.

Result S5: RNH domain stability is supported by PreR, related to Figure 1 and Figure 4

The first fork arm of PreR shows relatively more MSF than the second arm, which eventually gets minimized in the presence of RAG2 suggesting significant influence of its physical interaction (**Figure 1C**). The stability could be attributed to the PreR-RNH hydrophobic core, contributed by W522, P524, P525, L526, V529, and M685 (**Figure S3 A**), and strong H-bond between side chains of the residues S530 & R561 of RAG1, backbones of residues N528 of RAG1 & T168 of RAG2 and long-range electrostatic interaction between K527 of RAG1 (**Figure 4C**) and carbonyl oxygen of RAG2 T165 (**Figure S3 B**). The first fork arm of PreR is thus stabilized by hydrophobic as well as electrostatic interaction among residues.

There is a network of H-bond and other electrostatic interactions which also contribute to the stability of the PreR domain in the second arm of the fork. The residues D539 & K558 (β-sheet II and III) (**Figure S3 B**) make H-bond between their backbone as well as a sidechain, thus providing extra stability. The residue K558 also makes H-bond with E988 in the CTD domain. The residue R559 (β-sheet III) makes strong H-bond with E681 and weak electrostatic interaction with E170 of RAG2 (**Figure S3 B**). The residue R561 makes strong H-bond with D563 and S564 (**Figure S3 B**). The tip of the second arm, consisting of residue V548, is embedded in the RAG2 and is stabilized by hydrophobic interaction of I316, W317 and Y277 (**Figure 4D**). Therefore, it would be reasonable to believe that the second arm is also stabilized by electrostatic interactions along with hydrophobic interaction of the cylindrical hydrophobic core. However, the stalk region constitutes the largest proportion of Prer-RNH hydrophobic core (**Figure 4B**).

The RNH domain is comparatively stable and is primarily made up of β-sheets secondary structures. The domains PreR and RNH together form a cylindrical hydrophobic core wrapped over by hydrophilic residues (Figure 4A). The hydrophobic and electrostatic interactions are delicately balanced as most of the variants in our study lose their activity (Table S1 and Table S2). Besides the hydrophobically stabilized region of residue M605, there is an

electrostatically stabilized region, where RNH and CTD are held together around the residue R624. At this location, the residue R624 makes H-bond with E962 and electrostatic interaction with S626, H994, and E652 (**Figure S3 F**).

The RNH domain is very stable including the loop L34. Despite being deficient in secondary structure, stability is attained because of a complex network of short and long-range electrostatic interactions which engage the entire loop. The residues N650 and K648 of L34 make weak electrostatic interactions. However, S651, E652, and L653 are stabilized by H-bond interaction with the CTD domain (**Figure S3 G**). The H-bonding also plays a crucial role in stabilizing the interaction between α-helix I of RNH and RAG2, involving residues H668, E669 and A673 of RAG1 and Q16, K34, R73, N100 and N173 of RAG2 (**Figure S3 H**). Together these interactions stabilize RNH from the interior of the DNA-RAG1-RAG2 complex. The helix II of RNH defines its C-terminal and N-terminal of ZnC2. The helix II is near to the Zn binding region in ZnC2 which is stabilized by H-bond between R716 and carbonyl oxygen of C730 (**Figure S3 I**). Any destabilization in the region of RNH helix II will directly affect the sensitive Zn binding region of ZnC2.

Result S6: Three-sided interaction ZnC2 performs crucial role in stabilizing the complex, related to Figure 1 and Figure 2

The domain is primarily made of turns and coils for 62.5% of residues. However, three short helices (α-helix I R737-Q742, α-helix II H753-S765 and α-helix III V772-V779) (**Figure 1C**) also contribute to its stable architecture. The smaller fraction of residues involved in secondary structure formation makes the domain more labile and therefore it is supported by other domains to attain its functional conformation. The domain is exposed to solvents from one side and on the other side it is surrounded by ZnH2, RNH, and RAG2 (**Figure 2B**). The stability of the core is provided by a zinc atom held at the junction of ZnC2 and ZnH2 by C730 and C733 of ZnC2 and H940 and H945 of ZnH2 (**Figure 3D**). The CD of RAG2 plays a crucial role in shaping and stabilizing ZnC2 by providing residues for electrostatic as well as hydrophobic interactions. The hydrophobic residues in the hydrophobic cluster between α-helix II and α-helix III of ZnC2 interact with P37, P42 and Y68 of RAG2 (**Figure S3 J**). The electrostatic interaction supporting ZnC2 were observed among residues R776 and E722 of RAG1 and R39 of RAG2 from one side (**Figure S3 K**), while R764 of RAG1 with E126 and Y108 of RAG2 from the other side (**Figure S3 L**). The two helices are also supported by H-bond and salt-bridge formed among their solvent exposed residues. Y768, E770 and R778 (**Figure S4 A**). The domain is also hooked to RNH and ZnH2 through a group of hydrophobic residues in shape complementarily fashion (**Figure S3 G**). These stabilizing domain support makes it possible to place the residue R737 in right orientation for making strong electrostatic interaction as subsequently stabilizing the substrate DNA (**Figure S4 A**).

Result S7: Structural and functional characterization of ZnH2, related to Results section, Adjacent Zinc-Binding domains Coordinate with Cysteine (ZnC2) and Histidine (ZnH2)

ZnH2 is the domain following ZnC2 in RAG1. This domain is primarily made of α-helices (67.25 % of residues), no β-sheet and rest 32.75% of the residues are either coils or turns. The domain consists of eight helices, including short and long sequences, α-helix I is A796-G816, α-helix II is K826-M844, α-helix III is G854-L860, α-helix IV is K863-L872, α-helix V is E877-S898, α-helix VI is P906-L909, α-helix VII is Y912-K926, and α-helix VIII is N937-D953. The four long helices, α-helix I, V, VII and VIII, are nearly parallel to each other and make a cylindrical core. Other helices such as α-helix II, III and IV are stationed outside the central cylindrical structure and comprise most of the $RAG2^{Trans}$ interacting interface. Together these two groups of helices make the domain a typical globular protein with hydrophobic interior and polar and charged exterior. In the PRC complex, the major role of domain is to stabilize the ZnC2 and the substrate DNA near the heptamer region. The loop between the α-helix II and III is embedded in the minor grove with residues N853 and R851 and the residue K826 is facing the adjacent major groove (**Figure 3E**). However, its major role is to stabilize the nicked DNA strands and the trans RAG2 in HFC and STC.

The ZnH2 is among the most contributing domains for transition state stabilization. The characteristic feature of the domain is its α-helical composition and a long continuous hydrophobic core, which makes it more flexible than other domains. Because of the flexibility, the structure of the domain is slightly different than PRC in each state, with the RMSDs in NFC, HFC and STC being 1.189, 1.319 and 1.145 with respect to PRC, respectively. We observe side chain rearrangement in the domain during its transition between the states, which propels the helices to stabilize the nicked state of the DNA strand (**Video V1**).

Result S8: Structural and functional characterization of CTD, related to Figure 1 and Figure 3

The CTD consists of three helical regions (α-helix I D965-M977, α-helix II C984-T998, and α-helix III K1000-N1007). The first two helices of both RAG1 interact with both DNA. The third helix is separated by S999 and is placed in a slightly bent conformation leaning towards DDBD away from DNA (**Figure 1A**). The CTDs from two RAG1 chains do not interact with each other by hydrophobic or H-bond interaction; however, the coiled region on either side of the α-helix I of both the CTD has substantial interaction with both the DNAs (**Figure 3F and S6**). The positively charged residues in the α-helix I are placed at maximum distance from each other in the PRC with the terminals of the helix locked between the two DNAs. The distances between Cα and sidechain N atom in the residue K976 of both the CTD are 12 Å and 3.8 Å (**Figure S6** and **Video V2**). The two clusters of positively charged residues, K969, R972, R973 & R976, and R980 and K983, anchor the CTD of chain A with both the DNA from one side and another side of DNAs by the clusters of CTD of chain C. The residue R975 is projected away from the central region of the positively charged cluster but towards the loop L12, which is just above the CTD, and its role is to stabilize the loop in the PRC complex (**Figure S4 G**). The residues like L970, F974, V990, and Y997 from α-helix I and α-helix II respectively and the residue F478 from DDBD form a small hydrophobic cluster which stabilizes the CTD (**Figure S4 H**). The residues are partially or fully exposed to the

solvent except residue L1002. The residue is surrounded by hydrophobic environment made of residues L471, A472, L513 of DDBD and L996 of α-helix II of CTD. The residue M1006 also interacts partially with the hydrophobic region of the DDBD domain.

The N-terminal of CTD possesses an active site residue E965 and, along with the residue Q964, is projected in the minor groove of the substrate DNA. The residue S966 contributes to the stability of the active site residue and of loop L34 of RNH by making a strong H-bond with E652 (**Figure 3F and S3 G**). The positively charged residues like K969, R972, R973, R976, R980, and K983 and polar residues like Q981 and S982 are involved in the electrostatic stabilization of DNA. The functional role of these residues is further solidified by the loss of activity of variants R973C, R973H, and Q981P, to 0, 32, and 7.2 % (Table S1). Other residues in the α-helix I of the CTD domain that do not interact with DNA are F974 and R975, where F974 contributes to the stability by hydrophobic interaction and R975 by H-bond interactions with the loop L12 (**Figure S4 G and S4 H**). The variants F974L, R975Q, and R975W have 58.6%, 54.3%, and 40% of WT activity, respectively, which further corroborates our observations. The variant, K992E, with 10% activity, suggests that the long-range electrostatic interaction also plays a vital role in stabilizing DNA and CTD, where the N-atom of K992 is approximately 5Å away from the phosphate backbone oxygen atom of DNA.

The dynamic stability of α-helix III is also crucial for the activity of the complex; hydrophobic interactions comprise the primary stabilization. The loss in the activity of variant L1002F and gain in the activity of the variant M1006V suggests that non-bulky and small-sized residues, respectively, are comparatively more stable.

Supplemental Figures

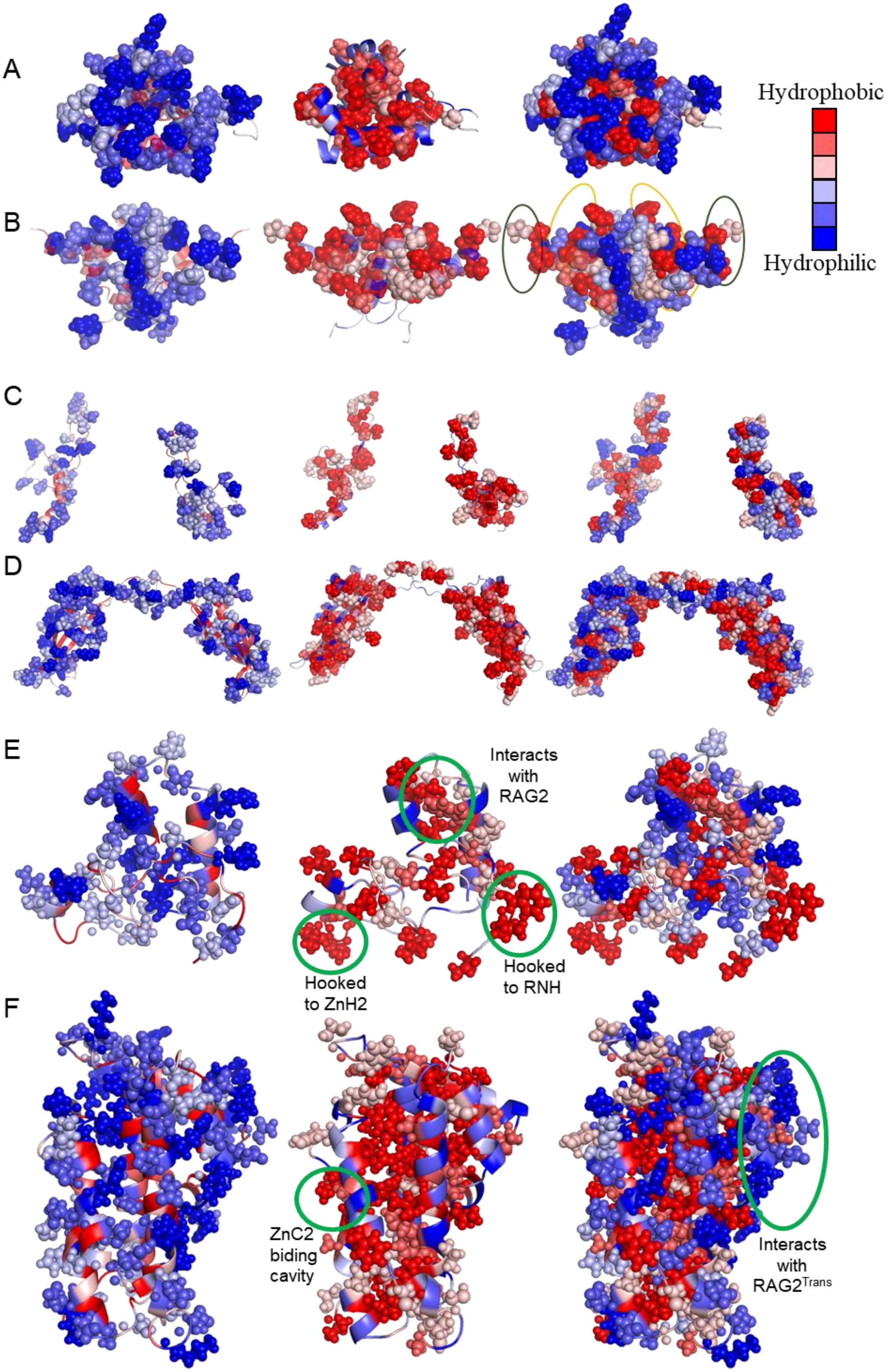

(caption on next page)

Figure S1 Hydrophobic residues spatial distribution, Related to Figure 1. Spatial distribution of sidechain of residues based on the hydrophobicity scale of Eisenberg (1984). The amino acids are classified into six groups based on their values on hydrophobic scale. They are (a) most hydrophobic (I, F, V & L), mild hydrophobic (W, M & A), least hydrophobic (G, C, Y and P), least hydrophilic (T, S & H), mild hydrophilic (E, N, Q & D) and most hydrophilic (K & R). Glycines are not shown on the backbone as they do not possess side chains. Figure (A) is NBD, (B) is DDBD. In the rightmost panel of "B", The yellow and green outlined region represents the presence of hydrophobic environment provided by CTD and Pre-R domain, respectively. Spatial distribution of hydrophobic and hydrophilic residues in PreR (C) and RNH (D). Patches of hydrophobic residues are observed on the surface their surfaces when estimated individually. Together they tend to make buried hydrophobic core (Figure 4A). (E) ZnC2 doesn't have a strict hydrophobic core. The domain is mostly stabilized by interdomain interactions, such as, RAG2, ZnH2 and RNH and Zn coordination near the ZnH2 domain. (F) ZnH2 is mostly helical and has a hydrophobic core. It interacts with RAG2^{trans} and stabilizes the HFC and STC states.

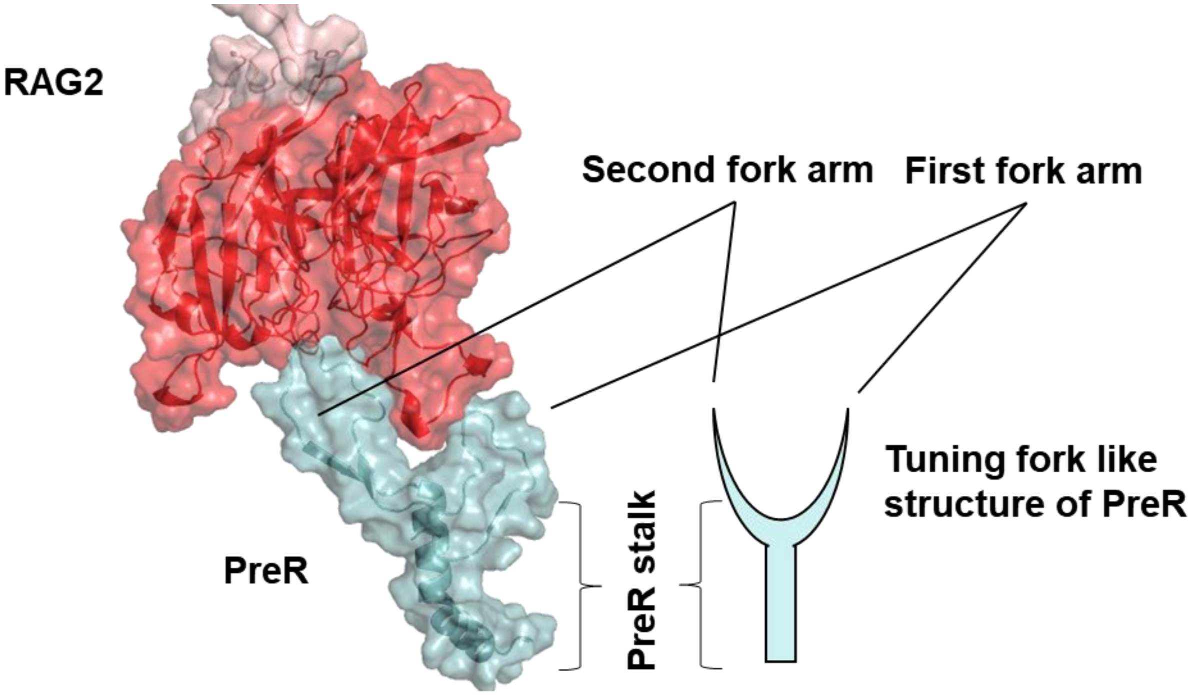

Figure S2: PreR strengthen the RAG1-RAG2 complex by stabilizing the loop of RAG2, Related to Figure 4. PreR, a tuning fork like structure, holds the RAG2 Y161-V175 loop between the two arms. Both electrostatic and hydrophobic interactions are involved in stabilizing the complex (Figure 4 B & C). First fork arm is from residue 520-534, second fork arm is from residue 536-561, and the PreR stalk is from 562-585 aa.

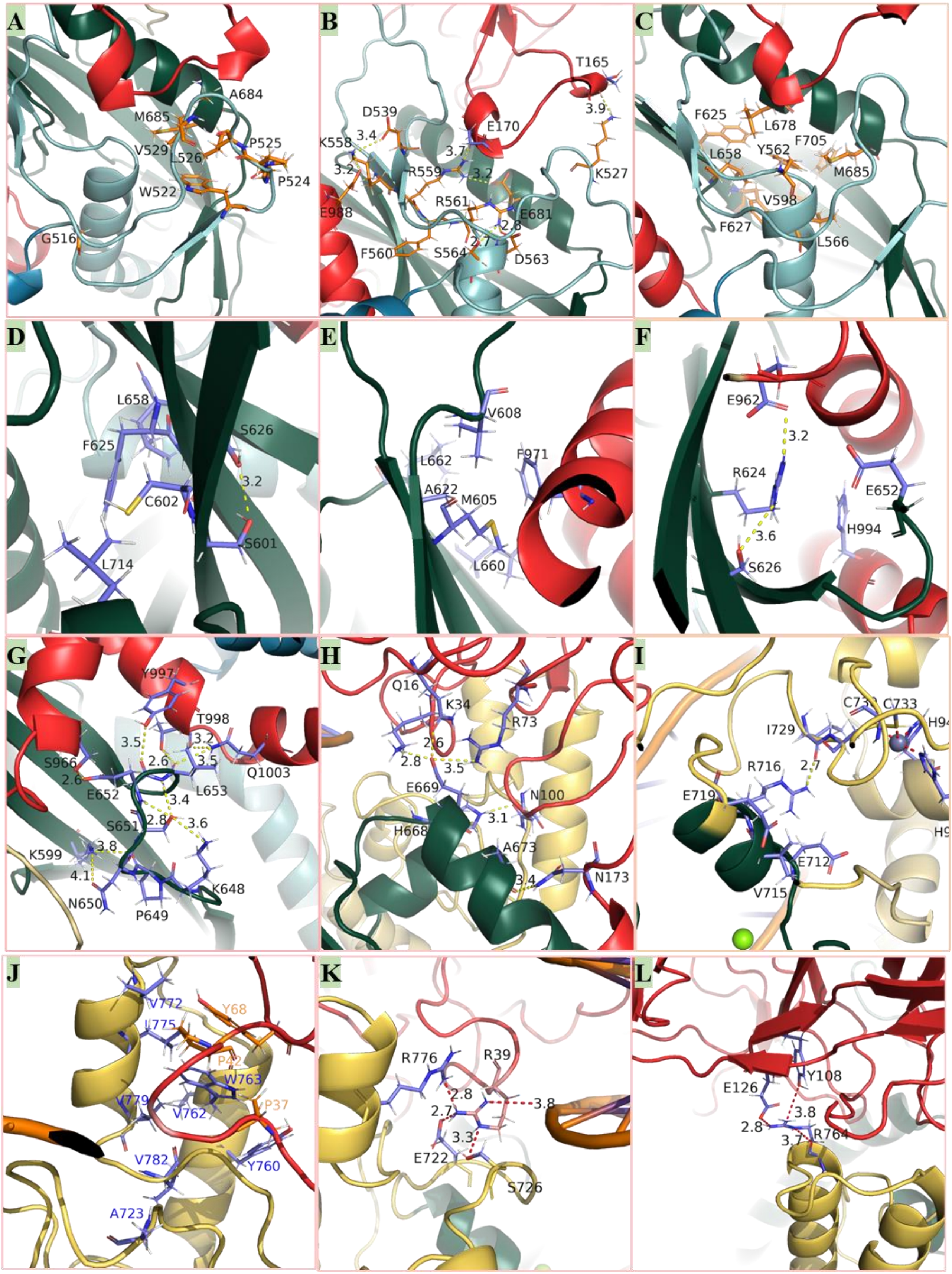

Figure S3: Interactions around PreR and ZNH domains, Related to Figure 3 and Figure 4. Zoomed in images majorly showing important interactions in PreR, ZNH and CTD domains. The details can be found in Table S3 and Table S4. The color of the domain is as shown in the Figure 1.

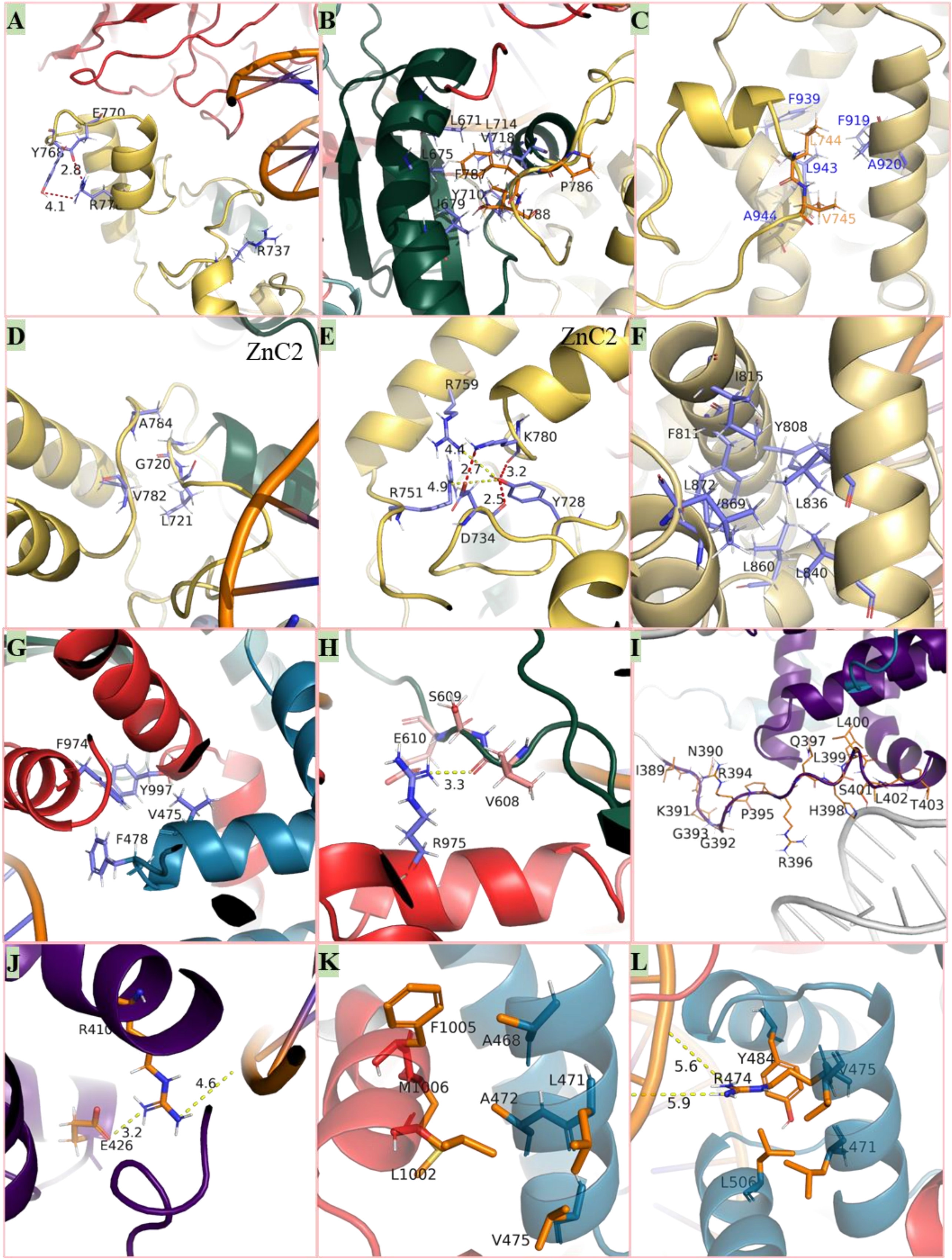

Figure S4: Interactions in ZnH2, ZnC2 and CTD domain, Related to Figure 3 and Figure 4. Zoomed in images showing important interactions in ZnH2, ZnC2 and CTD domains. The details can be found in Table S3 and Table S4. The color of the domain is as shown in the Figure 1.

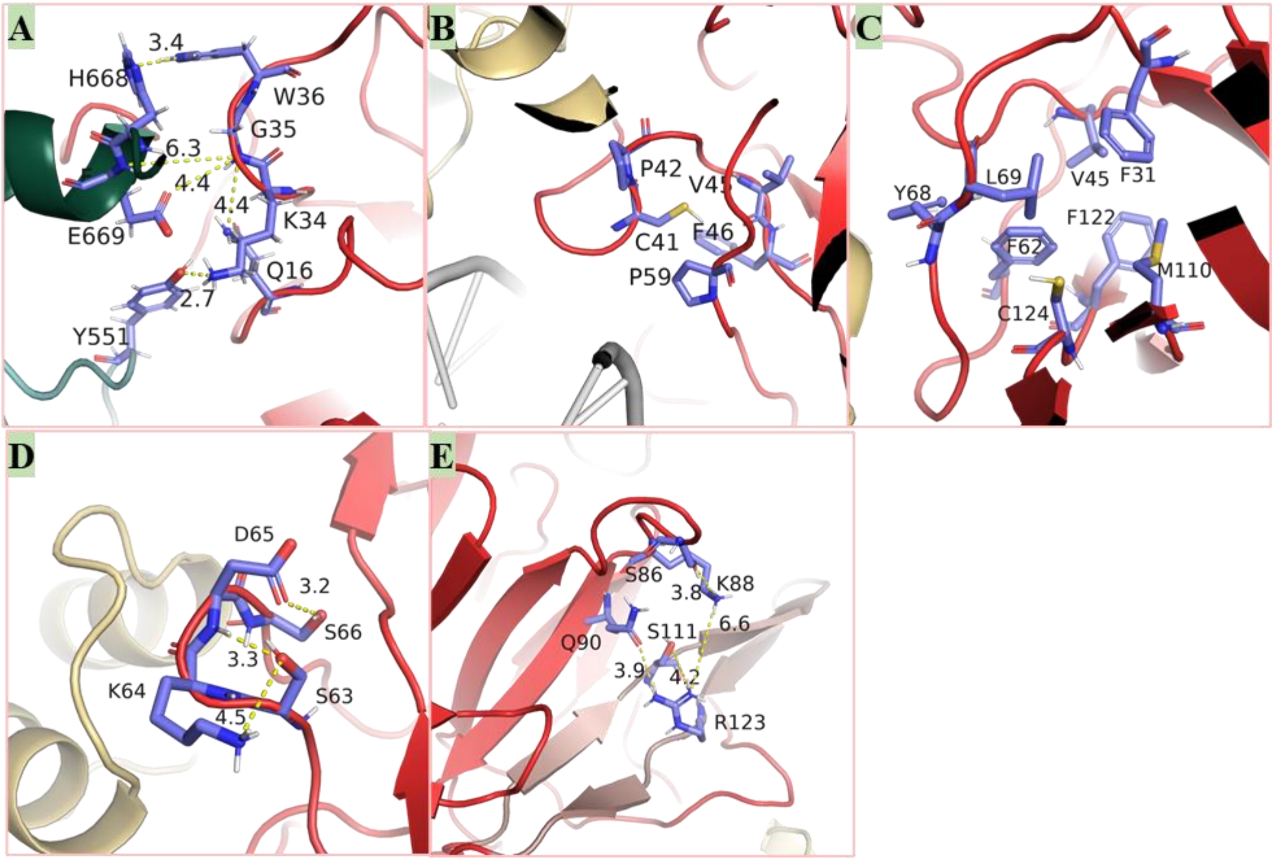

Figure S5: Interactions at RAG1/RAG2 interface, Related to Figure 3 and Figure 4. Zoomed in images showing important interactions in RAG1/RAG2 interface. The details can be found in Table S3 and Table S4. The color of the domain is as shown in the Figure 1.

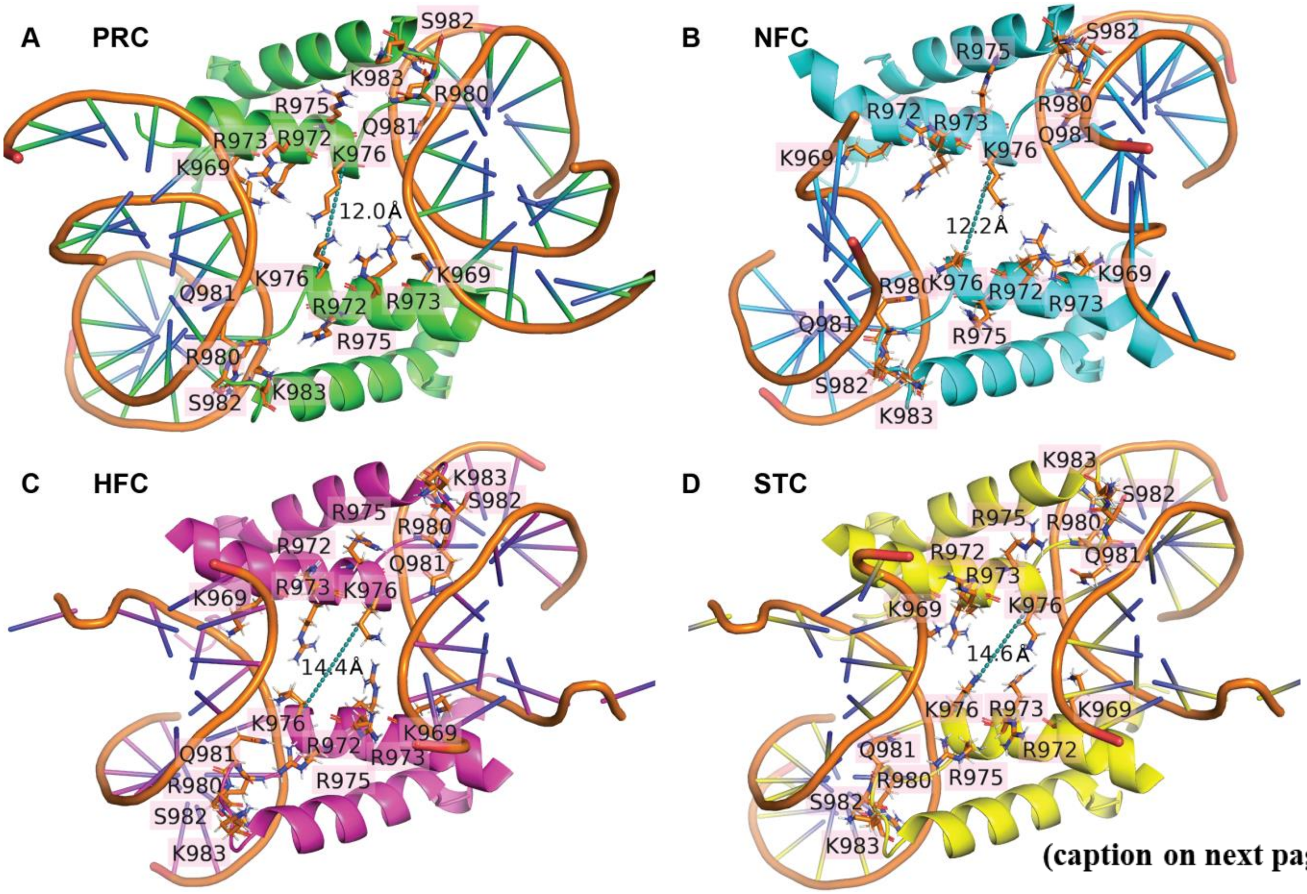

(caption on next page)

Figure S6: The CTD serves as a hinge point of rotation during RAG1/2 state change, Related to Figure 1 and Figure 2. The unique cluster of positively charged residues are held together at a specific distance by charge-charge repulsion while held with DNA by electrostatic interactions (**Figure 3F**). The rotation kind of motion around hinge can be observed by tracking distance between Cα-atom of residue K976 of both CTD units. A sequential increase of this distance 12.0, 12.2, 14.4, and 14.6 Å suggest increasing separation of the two CTD as it moves from PRC through STC.

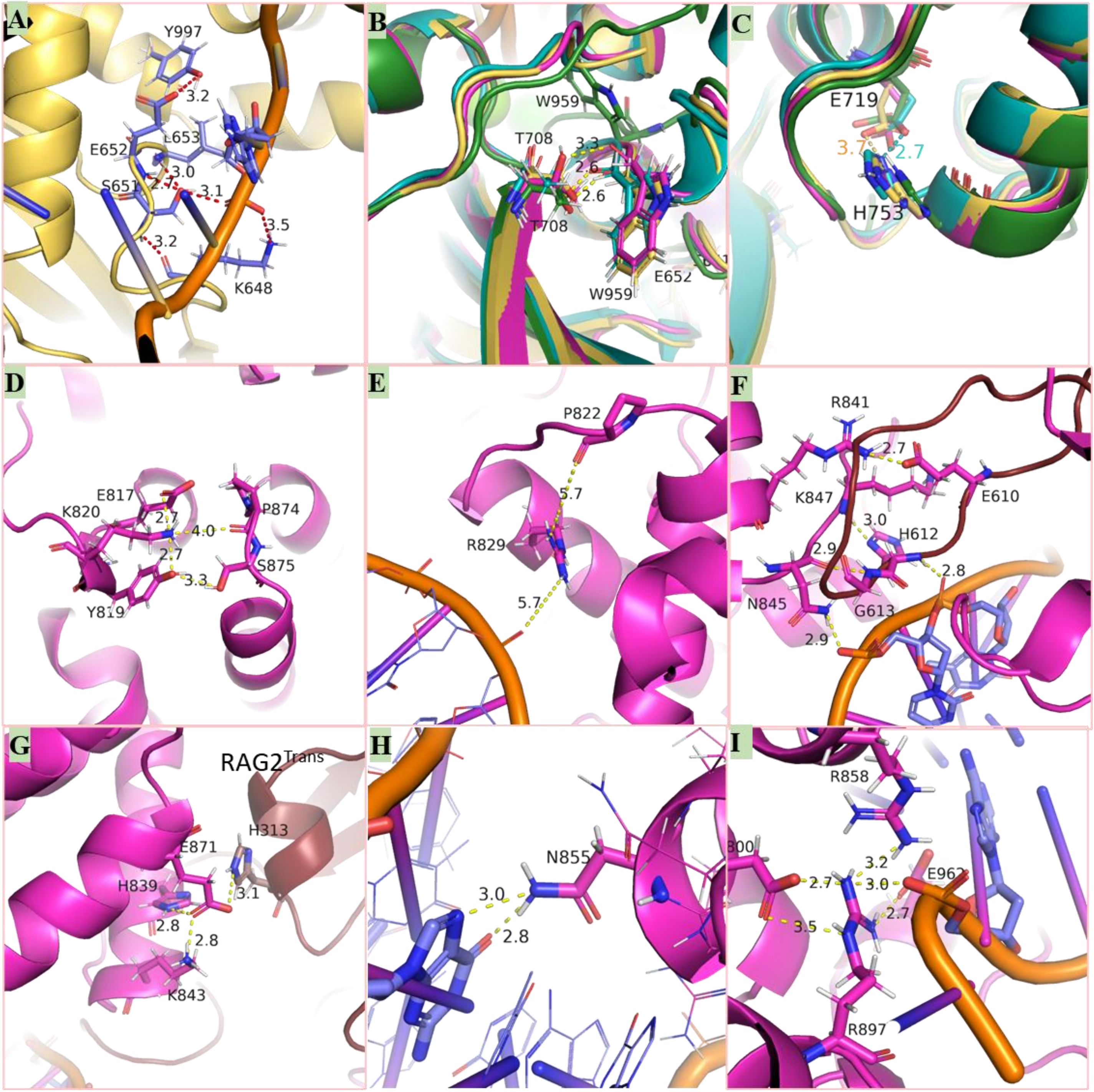

Figure S7: Intermediate of intermediate states, Related to Figure 1 and Figure 2: Important intermediate interactions from the state PRC (green), NFC (cyan), HFC (pink), and STC (yellow) are shown. The role of these interactions, A-I, are better understood by comparing the activity values of variants as discussed in Table S3, S651P, T708A, H753Q, K820R, R829S, R841Q, K843E, and R897Q.

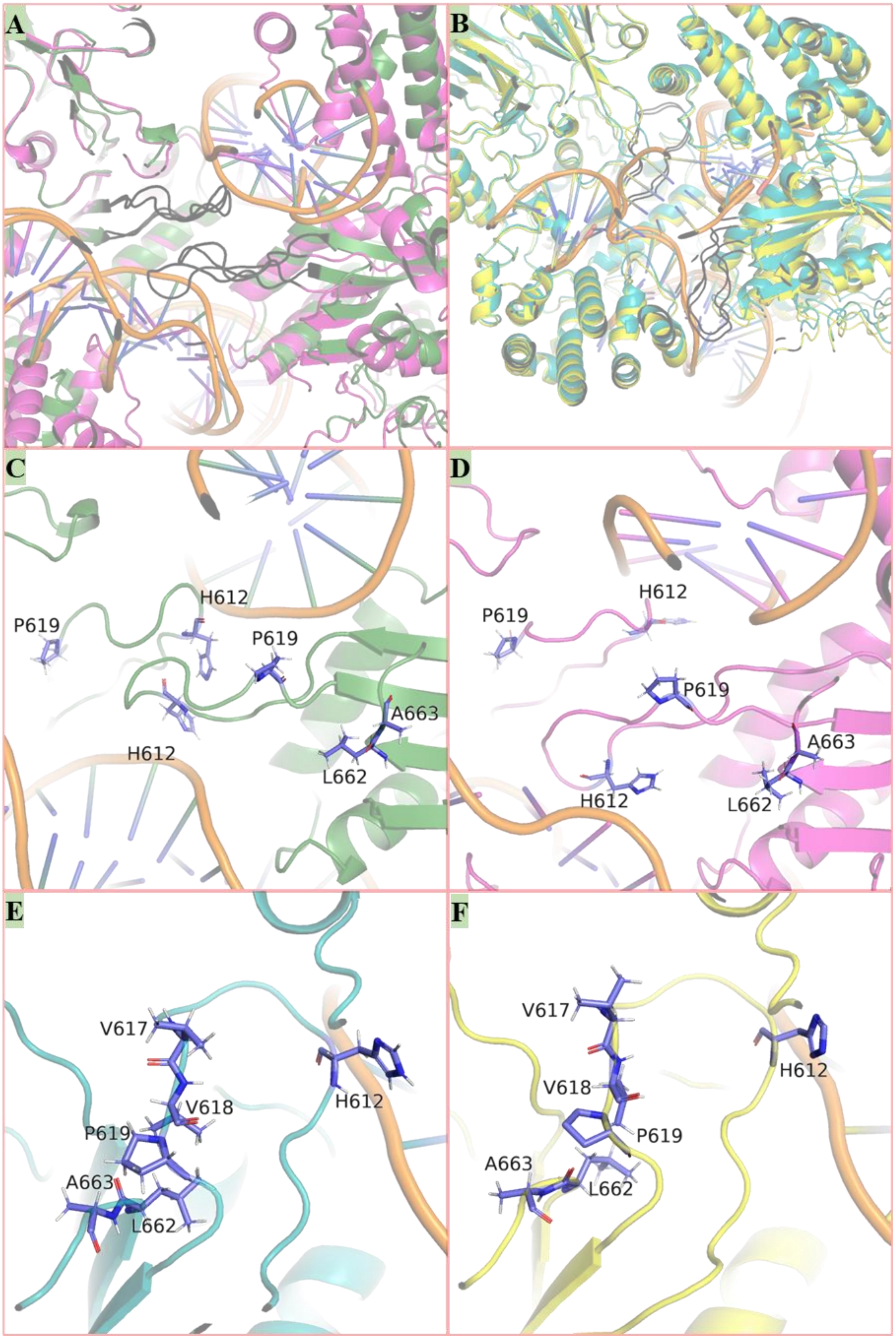

Figure S8: Spatial position of the L12 loop in different states of huDNA_RAG1_RAG2 complex during the recombinase activity, Related to Figure 1 and Figure 2. The black loop in the central region of Figure A (state PRC & NFC) and B (state NFC & STC). In the PRC and NFC state (Figure C&D respectively) the H612 is close to other DNA (trans interaction) and the P619 away from L662 and A663. In the HFC & STC state P619 comes close to the hydrophobic residues L662 and A663 (E & F).

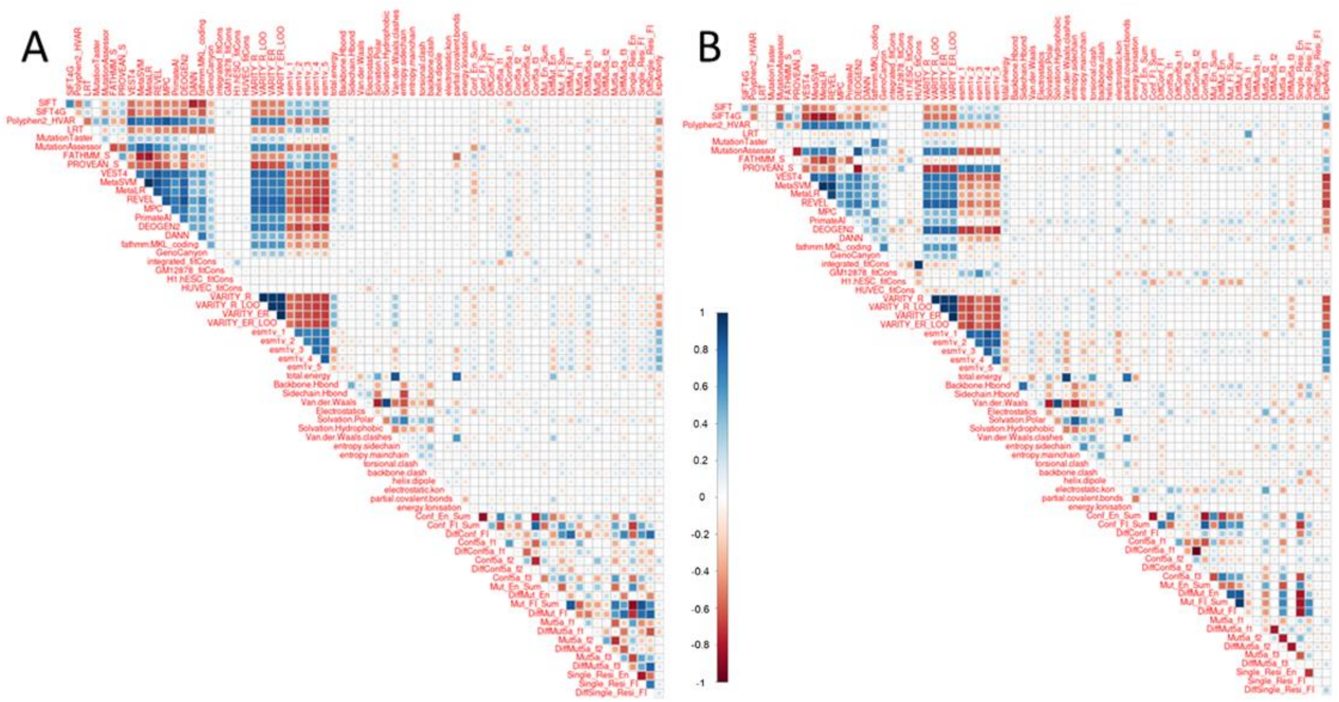

Figure S9: Protein sequence and structure-based scores form groups containing distinct information, Related to Figure 5. The correlation matrix of scores for A) RAG1 and B) RAG2 reveal a consistent relationship among scores. The sequence-based and protein language models are highly correlations with each other, while they are distinct from 3D calculations. Both types of scores correlate with experimental measurements. We interpret this observation that sequence-based scores account for the most severe mutations which have been selected against, while more subtle or context-dependent changes require 3D calculations to clarify the effects. The right-most column is experimental activity as measured in our *in vitro* system.

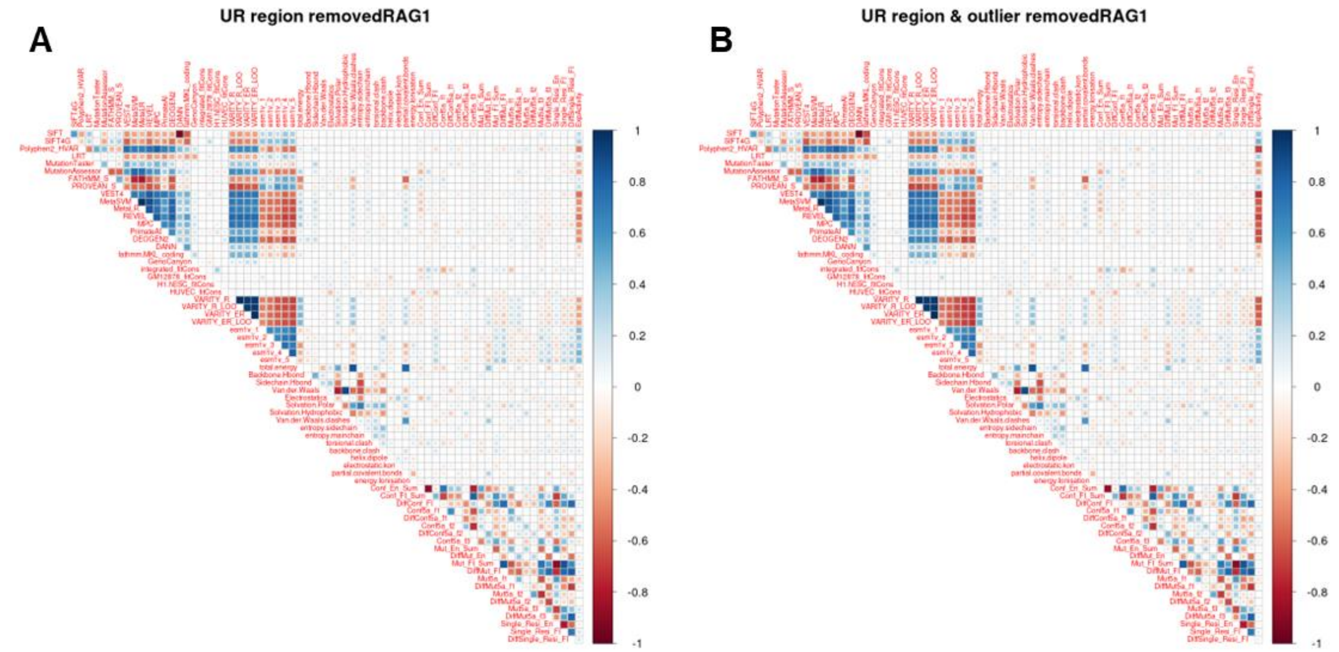

Figure S10: Removal of outliers and variants from unstructured region increases the correlation of scores with the experimental data in RAG1, Related to Figure 5. (A) Correlation plot after unstructured region removed. (B) Correlation plot after both unstructured region and outliers removed

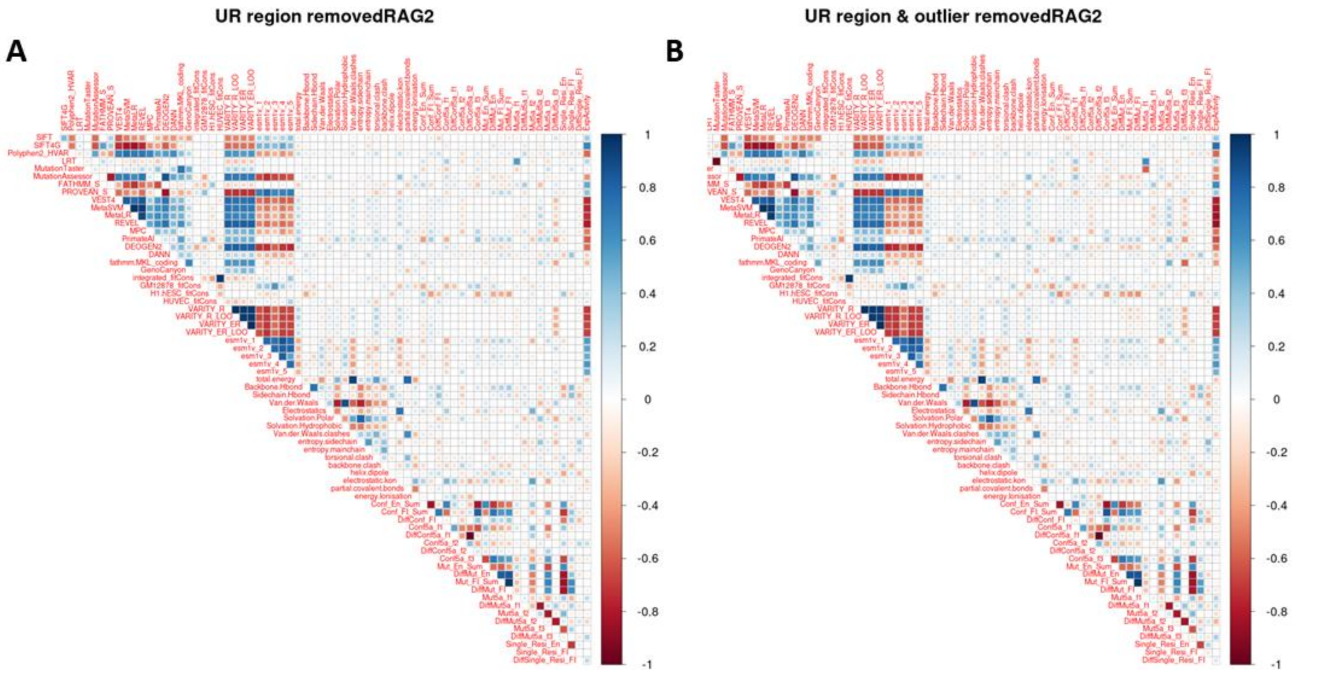

Figure S11: Removal of outliers and variants from unstructured region increases the correlation of scores with the experimental data in RAG2, Related to Figure 5. (A) Correlation plot after unstructured region removed. (B) Correlation plot after both unstructured region and outliers removed

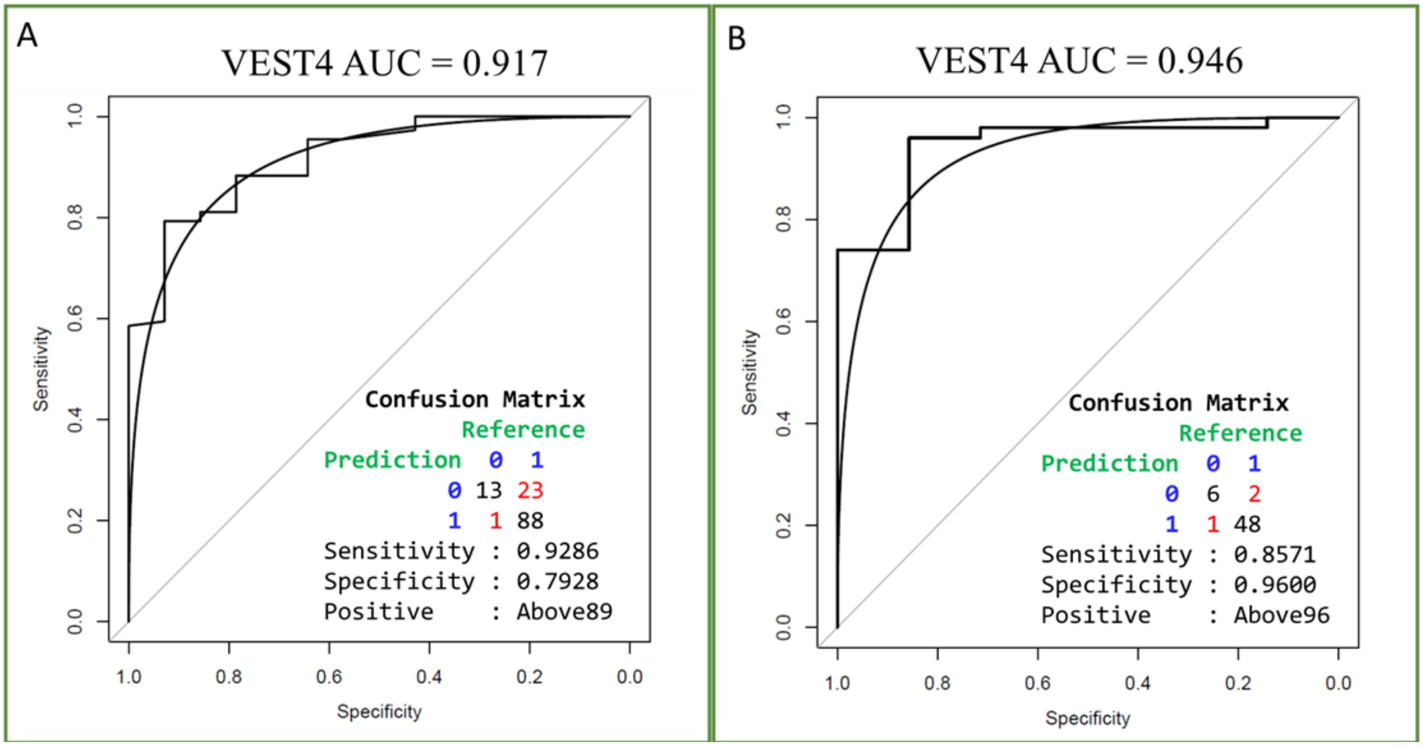

Figure S12: ROC-AUC plot and corresponding confusion matrix for two-class classification model using the pathogenicity prediction probability of VEST4 score, Related to Figure 5. A & B is ROC-AUC and confusion matrix for RAG1, RAG2 respectively.

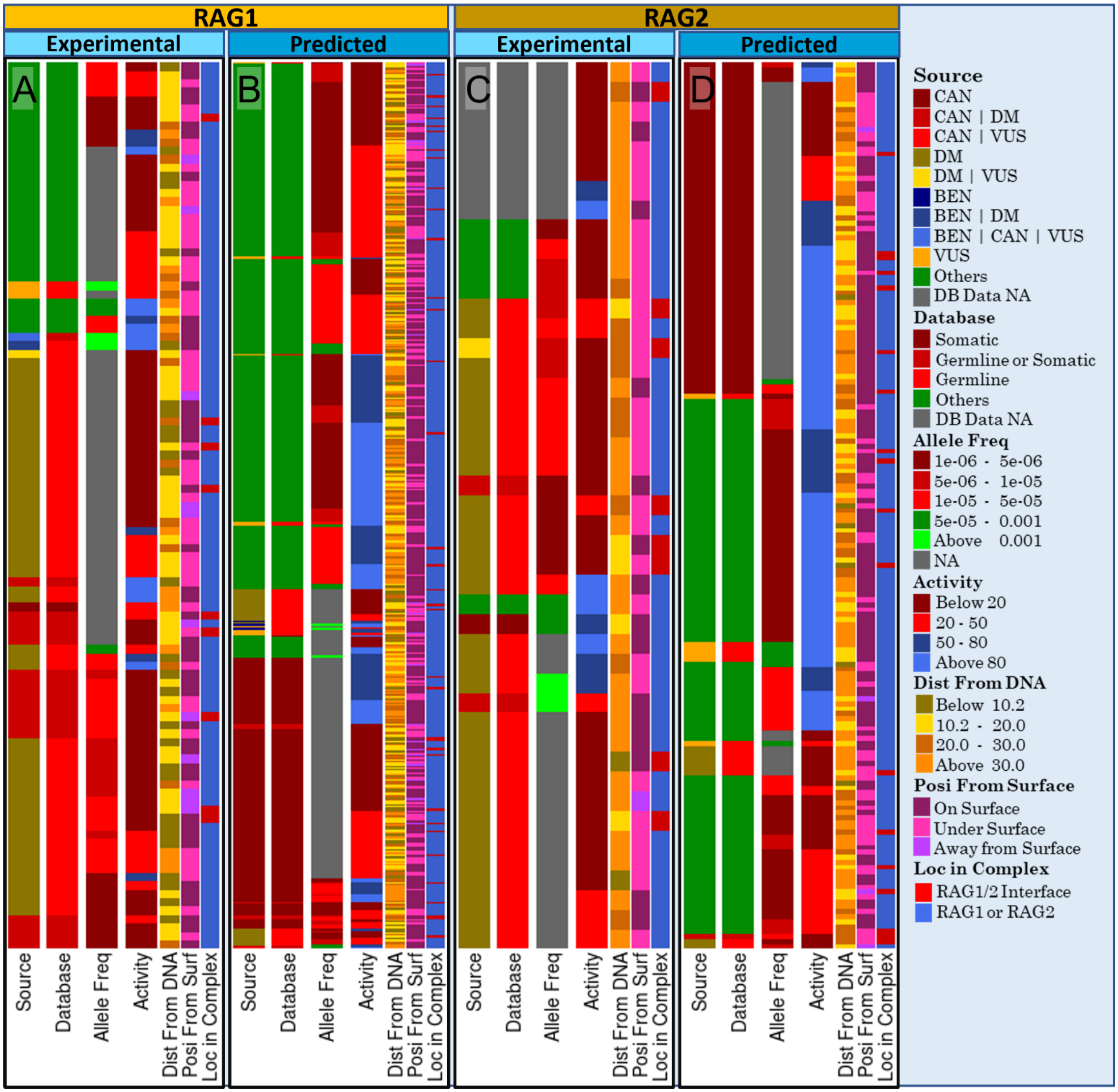

Figure S13: Heatmap of genomics, structural, and biochemical data of RAG1 and RAG2 variants reveals key patterns across IEI mutations that are shared by a fraction of population variations, Related to Figure 5 and Figure 6. The variant data is arranged as a set of four heatmap. "Experimental" refers to variants for which experimental data of enzymatic activity is available and the term "Predicted" refers to the variants for which experimental data is not available and their activity is predicted using the MLR model developed in the present work. where A) is RAG1 experimental, B) is RAG1 predicted, C) is RAG2 experimental, and D) RAG2 predicted. The term "experimental" and "predicted" are used for set of variants for which experimental activity data is available or they have been predicted using the model developed in the study, respectively. The first four columns of each heatmap are clustered using Euclidean distance metric of the variants. The last three columns of each heatmap are the annotation of clustered variants with their spatial location in the 3D model of RAG1/2-DNA complex. Columns of the heat map are defined as (i) Source, i.e., association of the variant with either somatic in cancer (CAN), germline disease mutant (DM), variant of uncertain significance (VUS), benign (BEN), healthy-population, others (Other), or no association record in databases (DB Data NA). (ii) Databases (DB), source of variant record, Somatic mutation DB, germline mutation DB, human population mutation and others (Others), or records with no DB information. (iii) Allele frequency, usually available in human mutation DB, (iv) Activity, experimental or predicted, (v), distance of variant from DNA in the 3D model in Angstrom, (vi), position of variant from the surface of the protein domain in the 3D model and (vi) location of the variant at the interface of RAG1 – RAG2 in the 3D model.

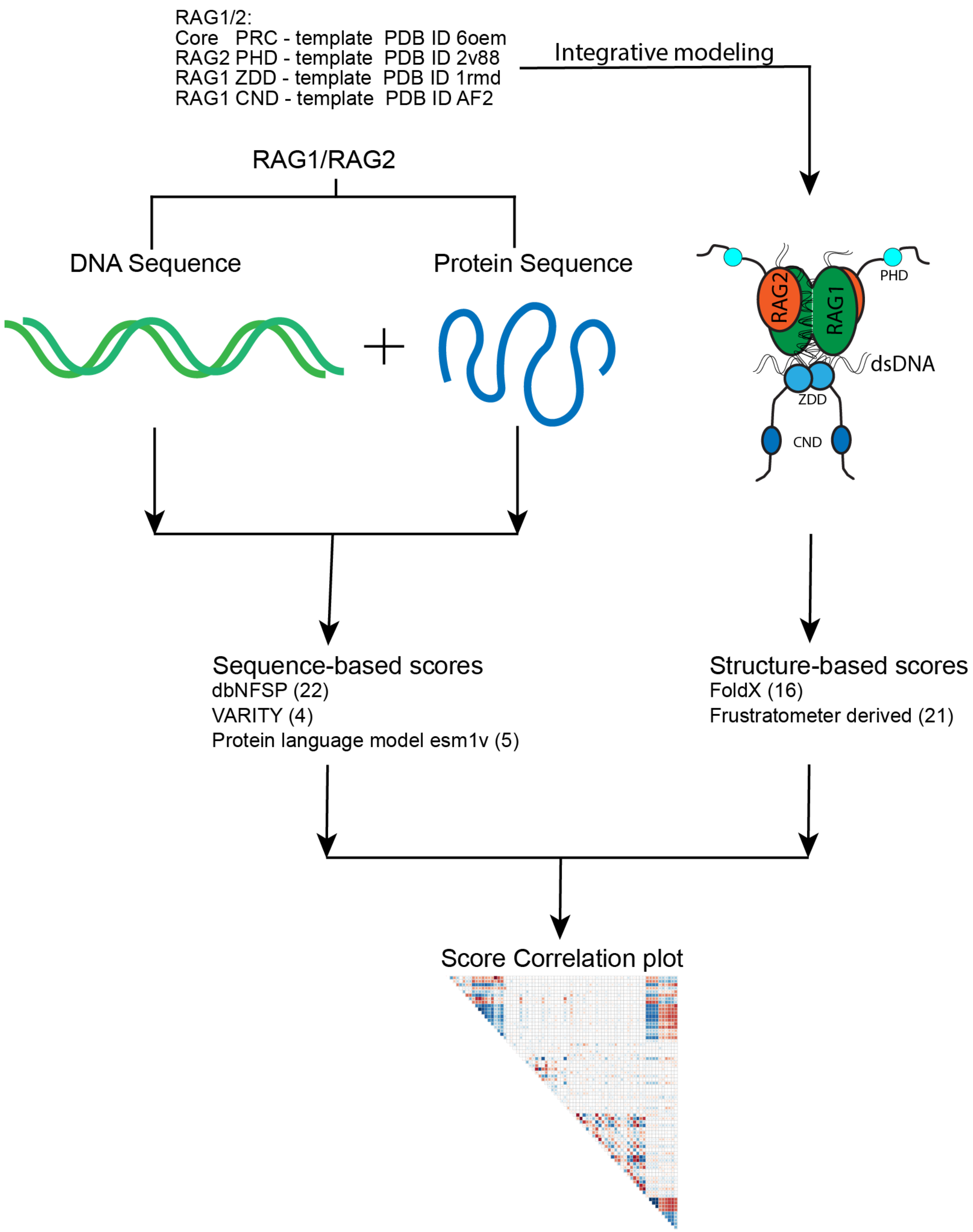

Figure S14: Workflow for scores generation, Related to Star Methods: A simplified flow representation of generation/computation of scores.

Table S5: Top correlated scores, Related to Figure 5. The Correlation of individual sequence- and structure-based scores with experimental measurements of recombinase activity

RAG1

	Score	CorSpear	CorPear
1	**VEST4_S**	0.436888835	**0.553875709**
2	REVEL_S	0.448706160	0.525231695
3	Polyphen2_HVAR_S	0.364069177	0.505061576
4	DEOGEN2_S	0.480768973	0.479598421
5	MPC_S	0.384828834	0.475027075
6	PrimateAI_S	0.438708979	0.464249703
7	**esm1v_t33_650M_UR90S_4.pt**	0.398313578	0.463735794
8	esm1v_t33_650M_UR90S_1.pt	0.407598762	0.444053891
9	MetaSVM_S	0.306037067	0.437177275
10	MetaLR_S	0.305796884	0.435693485
11	esm1v_t33_650M_UR90S_5.pt	0.368773989	0.417540611
12	esm1v_t33_650M_UR90S_2.pt	0.286653699	0.417373761
13	VARITY_R	0.323401068	0.406569755
14	VARITY_R_LOO	0.309987180	0.400794806
15	esm1v_t33_650M_UR90S_3.pt	0.311019950	0.397960548
16	fathmm.XF_coding_S	0.290576379	0.395029577
17	VARITY_ER	0.305318443	0.388104017
18	VARITY_ER_LOO	0.294654686	0.386020075
19	MutationAssessor_S	0.157404096	0.372266973
20	MutationTaster_S	0.319672465	0.333868787

RAG2

	Score	CorSpear	CorPear
1	**VEST4_S**	**0.55214108**	**0.7266942060**
2	REVEL_S	0.53506995	0.7121099412
3	VARITY_R	0.56129660	0.6560017593
4	MetaSVM_S	0.54238992	0.6534905215
**5**	**VARITY_R_LOO**	**0.58088880**	**0.6524229643**
6	**esm1v_t33_650M_UR90S_4.pt**	**0.64121099**	**0.6479821820**
7	MetaLR_S	0.57696235	0.6302413533
8	esm1v_t33_650M_UR90S_1.pt	0.58033776	0.6200645242
9	VARITY_ER_LOO	0.57657337	0.6196508286
10	MVP_S	0.48001686	0.6063767898
11	VARITY_ER	0.54225284	0.5991000924
12	esm1v_t33_650M_UR90S_2.pt	0.51823280	0.5805533013
13	esm1v_t33_650M_UR90S_5.pt	0.44912645	0.5524816970
14	PrimateAI_S	0.59579917	0.5426960282
15	SIFT4G_S	0.61410323	0.5384404082
16	esm1v_t33_650M_UR90S_3.pt	0.48925481	0.5233097806
17	DEOGEN2_S	0.30722853	0.5096149696
18	Polyphen2_HVAR_S	0.55219519	0.5017852525
19	MPC_S	0.45056726	0.4681928662
20	FATHMM_S	0.47853994	0.4456453809

Table S10 The distribution of variants in the RAG1/2 complex, Related to Figure 6B: Variants distributed on the surface, near the DNA and the interface of RAG1/RAG2 has been studied to characterize spatial location related behavior. The numbers in the table have been pictorially represented as bar plot in Figure 6B.

RAG1 Experimental (n = 105)
Count of variants distribution based on their location in the complex.

	NearDNA	OnSurf	IFace
AG1	18	26	8
AG2	5	11	1
AG3	2	3	0
AG4	3	5	0

RAG1 Experimental (n = 105)
Percentage of variants distribution based on their location in the complex.

	NearDNA	OnSurf	IFace
AG1	17.14	24.76	7.62
AG2	4.76	10.48	0.95
AG3	1.9	2.86	0
AG4	2.86	4.76	0

RAG1 Experimental (n = 532)
Count of variants distribution based on their location in the complex.

	NearDNA	OnSurf	IFace
AG1	40	86	17
AG2	33	92	13
AG3	8	78	5
AG4	5	93	3

RAG1 Experimental (n = 532)
Percent of variants distribution based on their location in the complex.

	NearDNA	OnSurf	IFace
AG1	7.52	16.17	3.2
AG2	6.2	17.29	2.44
AG3	1.5	14.66	0.94
AG4	0.94	17.48	0.56

RAG2 Experimental (n = 45)
Count of variants distribution based on their location in the complex.

	NearDNA	OnSurf	IFace
AG1	2	8	6
AG2	0	3	2
AG3	0	1	0
AG4	0	3	0

RAG2 Experimental (n = 45)
Percent of variants distribution based on their location in the complex.

	NearDNA	OnSurf	IFace
AG1	4.44	17.78	13.33
AG2	0	6.67	4.44
AG3	0	2.22	0
AG4	0	6.67	0

RAG2 Experimental (n = 179)
Count of variants distribution based on their location in the complex.

	NearDNA	OnSurf	IFace
AG1	1	10	5
AG2	0	17	3
AG3	0	17	2
AG4	6	72	8

RAG2 Experimental (n = 179)
Percent of variants distribution based on their location in the complex.

	NearDNA	OnSurf	IFace
AG1	0.56	5.59	2.79
AG2	0	9.5	1.68
AG3	0	9.5	1.12
AG4	3.35	40.22	4.47
